# Redox-Sensitive and Folate-Receptor-Mediated Targeting of Cervical Cancer Cells for Photodynamic Therapy Using Nanophotosensitizers Composed of Chlorin e6-Conjugated β-Cyclodextrin via Diselenide Linkage

**DOI:** 10.3390/cells10092190

**Published:** 2021-08-25

**Authors:** Howard Kim, Mi Woon Kim, Young-IL Jeong, Hoe Saeng Yang

**Affiliations:** 1 Department of Medicine, Graduate School, Dongguk University, Gyeongju 38067, Korea; howardkim83@gmail.com; 2 Department of Anesthesiology and Pain Medicine, College of Medicine, Dongguk University, Gyeongju 38067, Korea; swankim7@gmail.com; 3 Research Institute of Convergence of Biomedical Sciences, Pusan National University Yangsan Hospital, Gyeongnam 50612, Korea; 4 The Institute of Dental Science, Chosun University, Gwangju 61452, Korea; 5 Department of Obstetrics and Gynecology, Dongguk University College of Medicine, Gyeongju 38067, Korea

**Keywords:** photodynamic therapy, nanophotosensitizer, cervical cancer, ROS-sensitive, folate receptor

## Abstract

The aim of this study was to fabricate a reactive oxygen species (ROS)-sensitive and folate-receptor-targeted nanophotosensitizer for the efficient photodynamic therapy (PDT) of cervical carcinoma cells. Chlorin e6 (Ce6) as a model photosensitizer was conjugated with succinyl β-cyclodextrin via selenocystamine linkages. Folic acid (FA)-poly(ethylene glycol) (PEG) (FA-PEG) conjugates were attached to these conjugates and then FA-PEG-succinyl β-cyclodextrin-selenocystamine-Ce6 (FAPEGbCDseseCe6) conjugates were synthesized. Nanophotosensitizers of FaPEGbCDseseCe6 conjugates were fabricated using dialysis membrane. Nanophotosensitizers showed spherical shapes with small particle sizes. They were disintegrated in the presence of hydrogen peroxide (H_2_O_2_) and particle size distribution changed from monomodal distribution pattern to multimodal pattern. The fluorescence intensity and Ce6 release rate also increased due to the increase in H_2_O_2_ concentration, indicating that the nanophotosensitizers displayed ROS sensitivity. The Ce6 uptake ratio, ROS generation and cell cytotoxicity of the nanophotosensitizers were significantly higher than those of the Ce6 itself against HeLa cells in vitro. Furthermore, the nanophotosensitizers showed folate-receptor-specific delivery capacity and phototoxicity. The intracellular delivery of nanophotosensitizers was inhibited by folate receptor blocking, indicating that they have folate-receptor specificity in vitro and in vivo. Nanophotosensitizers showed higher efficiency in inhibition of tumor growth of HeLa cells in vivo compared to Ce6 alone. These results show that nanophotosensitizers of FaPEGbCDseseCe6 conjugates are promising candidates as PDT of cervical cancer.

## 1. Introduction

Cervical cancer, which is cancer that is derived from the cervix, is known to be the third- or fourth-most common cancer in women worldwide and the most common cancer in women in countries with low levels of resources [1,2]. Its incidence is caused by human papilloma virus infections in more than 90% of all cases [2,3]. Treatment of cervical cancer includes surgery, radiotherapy and chemotherapy according to the pathological stage [4,5,6]. Even though radical hysterectomy is believed to be one of the curative treatments for cervical cancer, its recurrence rate is more than 10% among the treated patients after radical surgery and it has less than a 5% 5-year survival rate [7,8,9]. Pre- or postoperative adjuvant therapy was tried to treat primary or recurred cervical cancer [10,11,12,13,14]. For example, Wang et al. reported that neoadjuvant chemotherapy of cervical cancers is effective in reducing the sizes of tumors and in potentiating the resectability of tumors [11]. These approaches may effectively reduce the risk of pathologic progress in cervical cancer. Kim et al. reported that postoperative adjuvant therapy with chemoradiation decreases the recurrence rate [12]. Chemotherapeutic agents effectively increased the median survival time of patients with cervical cancer, but the median survival time was still less than 20 months [15].

Photodynamic therapy (PDT) is defined as phototherapy of abnormal cells using photosensitizers and light sources. Since PDT is composed of light, photosensitizers and oxygen species are believed to be the safe candidates for cancer treatment [16,17,18]. PDT using photosensitizers does not significantly affect the viability of surrounding normal cells or tissues in the absence of light because photosensitizers are only activated in the irradiation field, producing ROS and thus killing the abnormal cells [16,17,18]. Furthermore, PDT would be an adequate option for squamous phenotype of tumor because the irradiation depth of tissues is less than 15 mm [17,18]. From these reasons, PDT is believed to be an efficient treatment option for cancers generated on the skin, epithelial tissue regions, or mucous layer [19,20,21,22,23]. For example, nanofiber mats incorporating 5-amino levulinic acid (5-ALA) are considered as a suitable device for PDT of bile duct cancer because cancer cells in bile duct are frequently spread out through the bile duct walls [19]. Chlorin e6 (Ce6)-based PDT is also believed to be a promising candidate for imaging and treatment of colon cancer cells [21]. Furthermore, the morphological composition of cervical cancers is predominantly squamous cell carcinoma, which is about 90%, while the other 10% is adenocarcinoma [3,4]. In this viewpoint, PDT can be also considered as a plausible adjuvant therapy for cervical cancers and cervical disorders [24,25,26]. Wierrani et al., reported that 5-ALA-mediated PDT resulted in 80% of ectocervical dysplasia and therefore, concluded that PDT has efficacies to treat ectocervical dysplasia [25]. Furthermore, Trushina et al., reported that PDT induces complete regression of 94.2% of pre-cancerous lesions and 83.4% of non-invasive cervical cancer [26]. In spite of its potential fighting against cervical cancer, PDT still has problems in practical application. For example, repeated PDT of tumor using 5-ALA induces oral squamous cell carcinoma to be resistant against photosensitizer [27]. Even though photosensitizers predominantly accumulate in the cancer cells, traditional photosensitizers normally spread out through the whole body after systemic administration due to their low specificity against cancer cells [28]. These problems induce necessity of protection from sunlight for a while [29]. Also, low aqueous solubility of photosensitizers is also problematic for clinical application [30]. Therefore, novel vehicles should be developed to solve these problems.

Various kinds of devices or materials such as polymers, nanomaterials, natural polysaccharide and cyclodextrins have been investigated to solve drawbacks of photosensitizer through tissue-specific delivery [30,31,32,33,34,35,36]. For example, Ce6-incorporated nanophotosensitizers showed improved cellular uptake, higher phototoxicity and ROS generation against cholangiocarcinoma cells [32]. Furthermore, Ce6-chitosan nanocomplexes were absorbed efficiently through the mucosal layer of porcine bile duct explants compared to Ce6 alone [33]. Colloidal nanocarriers for PDT of cervical cancer have minimal toxic side-effects and improved patient survival rates [34]. Also, cyclodextrins have been employed to solve aqueous solubility of photosensitizers and to improve PDT efficacy [35]. Ben Mihoub et al., reported that inclusion complexes using β-cyclodextrin enhance aqueous solubility and phototoxicity of porphyrin derivatives [36]. Furthermore, cyclodextrins are known to enhance drug permeability through biomembranes such as mucous layer [37].

In this study, we synthesized ROS-sensitive nanophotosensitizers composed of β-cyclodextrin, Ce6 and MePEG-folate via diselenide linkage (FaPEGbCDseseCe6) for folate receptor/ROS-sensitive targeting of photosensitizers. Physicochemical properties of FaPEGbCD-sese-Ce6 nanophotosensitizers were investigated in vitro. Furthermore, PDT efficacy of FaPEGbCD-sese-Ce6 nanophotosensitizers was evaluated in vitro and in vivo tumor xenograft model using HeLa cells.

## 2. Materials and Methods

### 2.1. Chemicals

Ce6 was purchased from Frontier Sci. Co. (Logan, UT, USA). Folic acid, succinyl β-cyclodextrin, 2’,7´-dichlorofluorescin diacetate (DCFH-DA), hydrogen peroxide (H_2_O_2_), N-(3-dimethylaminopropyl)-N’-ethylcarbodiimide hydrochloride (EDAC), N-hydroxy succinimide (NHS), 3-(4,5-dimethyl-2-thiazolyl)-2,5-diphenyl-2H-tetrazolium bromide (MTT), selenocystamine dihydrochloride and Cremophor^®^ EL were purchased from Sigma Aldrich Chem. Co. (St. Louis, MO, USA). Poly(ethylene glycol)-diamine (molecular weight: 2000 g/mol) was purchased from SunBio Co. Ltd. (Seoul, Korea). Dialysis membranes with molecular weight cutoffs size (MWCO) of 500, 2000 and 8000 g/mol were purchased from Spectra/Pro^TM^ Membranes.

### 2.2. Synthesis of FaPEGbCDseseCe6 Conjugates

Ce6-selenocystamine conjugates: Ce6 (478 mg) dissolved in 10 mL DMSO was mixed with equivalent moles of EDAC and NHS and then magnetically stirred for 3 h. To this solution, three equivalent moles of selenocystamine dihydrochloride (762 mg) dissolved into 20 mL of DMSO/water mixtures (9/1, *v/v*) were added with two equivalent moles of TEA and then magnetically stirred for more than 12 h. Following this, the resulting solution was introduced into a dialysis tube (MWCO = 500 g/mol) and then dialyzed against water for 2 days. The water was exchanged every 3 h intervals to remove organic solvents and byproducts. Following this, the dialyzed solution was lyophilized over 2 days. 

Succinyl β-cyclodextrin-selenocystamine-Ce6 conjugates: Succinyl β-cyclodextrin (183 mg, 0.1 mM) was dissolved into 20 mL of DMSO with 7 equivalent moles of EDAC and NHS. This solution was magnetically stirred for 6 h. After that, 578 mg of Ce6-selenocystamine conjugates were added to this solution. This was further stirred for 24 h and then the resulting solution was introduced into a dialysis tube (MWCO = 2000 g/mol) and dialyzed against water for 2 days. The water was exchanged every 3 h intervals to remove organic solvents and byproducts. The resulting solution was lyophilized over 2 days to obtain succinyl β-cyclodextrin-selenocystamine-Ce6 conjugates. The yield of the products was calculated from mass measurement as follows: yield = [weight of lyophilized product/(weight of succinyl β-cyclodextrin + weight of Ce6-selenocystamine conjugates)] × 100. The yield of succinyl β-cyclodextrin-selenocystamine-Ce6 conjugates was higher than 92% (*w/w*). 

FA-PEG conjugates: 133 mg of folic acid were dissolved in 20 mL of DMSO with equivalent mole of EDAC and NHS. To this solution, 600 mg of poly(ethylene glycol)-diamine were added and magnetically stirred for 24 h. Following this, the resulting solution was introduced into a dialysis tube (MWCO = 2000 g/mol) and then dialyzed against water for 2 days. Water was exchanged every 3 h intervals to remove organic solvents and byproducts. The resulting solution was lyophilized over 2 days to obtain FA-PEG conjugates.

FaPEGbCDseseCe6 conjugates: 150 mg of succinyl β-cyclodextrin-selenocystamine-Ce6 conjugates were dissolved into 20 mL of DMSO. To this solution, 12 mg of EDAC and 7 mg of NHS were added and then magnetically stirred for 6 h. After that, 146 mg of FA-PEG conjugates were added and then magnetically stirred for more than 36 h. Following this, the resulting solution was introduced into a dialysis tube (MWCO = 8000 g/mol) and then dialyzed against water for 2 days. The water was exchanged every 3 h to remove organic solvents and byproducts. The resulting solution was lyophilized over 2 days to obtain FaPEGbCDseseCe6 conjugates. 

The yield of the final products was calculated from mass measurements as follows: yield = [weight of lyophilized product/[(weight of succinyl β-cyclodextrin-selenocystamine-Ce6 conjugates + weight of FA-PEG conjugates)] × 100. The yield of the FaPEGbCDseseCe6 conjugates was higher than 94% (*w/w*). Based on the mass measurements, the conjugation number of FA-PEG in the FaPEGbCDseseCe6 conjugates was approximately 2.8 FA-PEG/ FaPEGbCDseseCe6 conjugates. 

For comparison, FaPEGbCD conjugates were also synthesized as follows: succinyl β-cyclodextrin (37 mg) was dissolved into 10 mL of DMSO. To this solution, 12 mg of EDAC and 7 mg of NHS were added and then magnetically stirred for 6 h. After that, 146 mg of FA-PEG conjugates were added and then magnetically stirred for more than 36 h. Following this, the resulting solution was introduced into a dialysis tube (MWCO = 8000 g/mol) and then dialyzed against water for 2 days. Water was exchanged every 3 h to remove organic solvents and byproducts. The resulting solution was lyophilized over 2 days to obtain FaPEGbCD conjugates. These were used as empty nanophotosensitizers.

### 2.3. ^1^H Nuclear Magnetic Resonance (NMR) Spectra

^1^H NMR spectra (500 mHz superconducting Fourier transform (FT)-NMR spectrometer, Varian Unity Inova 500 MHz NB High-Resolution FT NMR; Varian Inc., Santa Clara, CA) was used to monitor the synthesis of conjugates. To measure the ^1^H NMR spectra, conjugates were dissolved in D_2_O, DMSO or D_2_O/DMSO mixtures.

### 2.4. Fabrication of FaPEGbCDseseCe6 Nanophotosensitizers 

FaPEGbCDseseCe6 conjugates (20 mg) dissolved in 5 mL DMSO/water mixtures (4/1 *v*/*v*) were introduced into the dialysis membrane (MWCO = 8000 g/mol). A dialysis membrane was placed into a beaker with 1 L of deionized water and dialyzed for 1 day. To prevent saturation of organic solvent, deionized water was exchanged every 3 h for 24 h and, after that, the resulting solution was adopted to analyze or to evaluate the PDT effect. The Ce6 contents were evaluated using the following method: FaPEGbCDseseCe6 (5 mg) was reconstituted into 50 mL phosphate-buffered saline (PBS, 0.01 M, pH 7.4) and then H_2_O_2_ was added (the final concentration of H_2_O_2_ was 20 mM). This was stirred for more than 48 h and, after that, it was diluted with DMSO by 10 times. Fluorescence spectrophotometer (excitation wavelength: 407, emission wavelength: 664 nm) (RF-5301PC spectrofluorophometer, Kyoto, Japan) was used to measure Ce6 concentration and then calculated Ce6 contents in the FaPEGbCDseseCe6. For comparison, free Ce6 dissolved in DMSO was diluted with phosphate-buffered saline (PBS, 0.01 mM pH 7.4) and then added H_2_O_2_ (The final concentration of H_2_O_2_ was 20 mM).

Ce6 content (wt.%) = (Ce6 weight/total weight of nanophotosensitizers)/100.

Ce6 content in the FaPEGbCDseseCe6 was 19.2% (*w/w*).

### 2.5. Transmission Electron Microscope 

Transmission electron microscopy (TEM) (H-7600, Hitachi Instruments Ltd., Tokyo, Japan) was used to observe the morphology of the FaPEGbCDseseCe6 nanophotosensitizers. One drop of the nanophotosensitizer solution was placed onto the carbon-film-coated grid and then dried at room temperature. The observation was carried out at 80 kV.

### 2.6. Fluorescence Spectrophotometer Measurement 

To measure the fluorescence properties, FaPEGbCDseseCe6 nanophotosensitizers fabricated as described above were diluted with PBS and then the Ce6 concentration was adjusted to 0.1 mg/mL in PBS with or without H_2_O_2_. This solution was incubated for 4 h at 37 °C to react with H_2_O_2_. Then, the resulting solutions were scanned using a fluorescence spectrofluorophotometer (RF-5301PC Spectrofluorophometer, Kyoto, Japan). Nanophotosensitizers were adopted to scan the emissions between 500 and 800 nm (excitation wavelength: 400 nm). The fluorescence image of this solution was also measured with a Maestro 2 small animal imaging instrument (Cambridge Research and Instrumentation Inc., Woburn, MA 01801, USA).

### 2.7. Drug Release from Nanophotosensitizer 

The nanophotosensitizers fabricated as described above were used to study the drug release in PBS (0.01 M, pH 7.4) in the presence of H_2_O_2_. The concentration of nanophotosensitizer solution was adjusted to 20 mg nanophotosensitizers/20 mL of water. Then, this solution (5 mL) was introduced into the dialysis membrane (MWCO = 8000 g/mol). After that, the dialysis membrane was put into a conical tube with PBS (45 mL, pH 7.4, 0.01 M). H_2_O_2_ was added to investigate the ROS-sensitive drug release from the nanophotosensitizers. The nanophotosensitizer solution was incubated at 37 °C and 100 rpm with a shaker incubator (SI-600R, Jeiotech Co., Daejeon, Korea). The whole media was taken to measure the Ce6 concentration in the release media and then fresh PBS was added to continue the drug release study. Ce6 concentration in the release media was measured with a fluorescence spectrofluorophotometer (RF-5301PC Spectrofluorophotometer, Kyoto, Japan) (excitation wavelength: 407, emission wavelength: 664 nm). All of the results are expressed as mean ± standard deviation (S.D.) from triplicated experiments.

### 2.8. Cell Culture

HeLa human cervical cancer cells and CCD986sk human skin fibroblast cells were purchased from Korean Cell Line Bank (Seoul, Korea). HeLa cells were maintained in an MEM medium (Gibco, Grand Island, NY, USA), supplemented with 10% heat-inactivated fetal bovine serum (FBS) (Invitrogen) and 1% penicillin/streptomycin at 37 °C in a 5% CO_2_ incubator. CCD986sk cells were maintained using IMDM (Gibco, Grand Island, NY, USA) supplemented with 10% heat-inactivated fetal bovine serum (FBS) (Invitrogen) and 1% penicillin/streptomycin.

### 2.9. Light Source for PDT Treatment

**Scheme 1:**
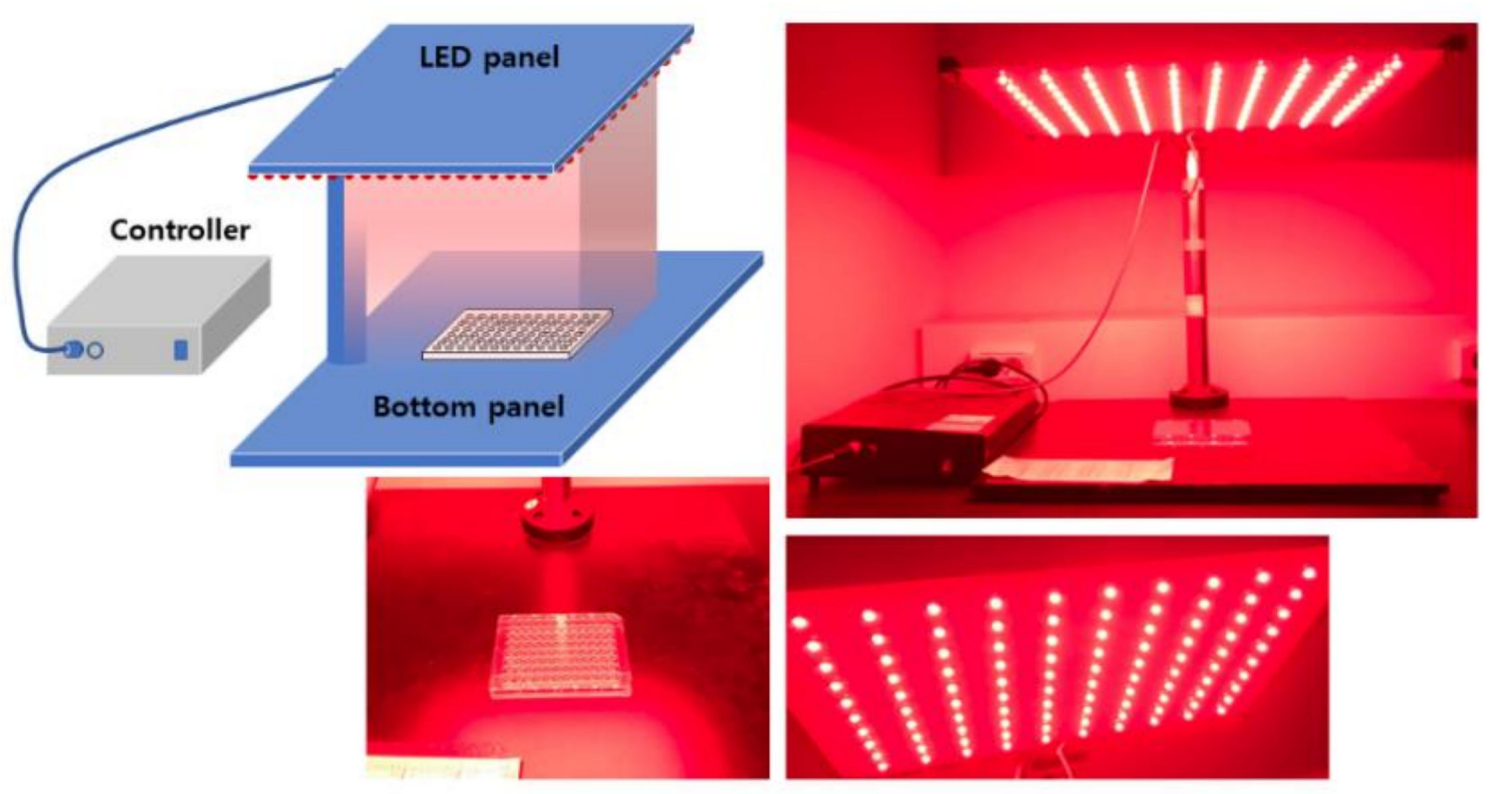
Light source that was used for the PDT of cancer cells.

An expanded homogenous beam (SH Systems, Gwangju, Korea) was used to perform PDT of cells at 664 nm (2.0 J/cm^2^) [38]. The distance between panel having LED source from the cells in the well plate was 40 cm, as shown in Scheme 1. Cells in the 96 well plate were located in the center of the bottom panel. To determine the light dose, photo-radiometer (DeltaOhm, Padova, Italy) was used to measure the signal of light. We measured the light intensity at more than 20 points and the light dose was calculated in the center of the bottom panel.

### 2.10. PDT Treatment of Cancer Cells 

Phototoxicity: HeLa cells (2 × 10^4^ cells/well) were seeded in 96-well plates and cultured overnight in 5% CO_2_ at 37 °C. These cells were then treated with either the Ce6 or FaPEGbCDseseCe6 nanophotosensitizers. For the Ce6 treatment, the Ce6 dissolved in DMSO was diluted by more than 100 times with serum-free culture media. The FaPEGbCDseseCe6 nanophotosensitizers in an aqueous solution were also diluted with serum-free culture media. 2hr after the treatment, these cells were washed twice with PBS (pH 7.4, 0.01 M) and then 100 µL of serum-free and then phenol red-free MEM media were added. For the PDT treatment, cells were irradiated with an expanded homogenous beam at 664 nm (2.0 J/cm^2^). The light dose was measured with a photo-radiometer (DeltaOhm, Padova, Italy). The cells were incubated for 24 h in a CO_2_ incubator at 37 °C in the dark to avoid visible light. Following this, the cell viability was measured with an MTT proliferation assay: 30 µL MTT solution (5 mg/mL in PBS) was added to the cultured cells and then these were further incubated for 4 h at 5% CO_2_. After that, DMSO (100 µL) was added to the cells and agitated at 50 rpm in a shaker incubator (37 °C) for 10 min. These were measured with an Infinite M200 Pro microplate reader at 570 nm. All procedures were performed in the dark condition.

Dark toxicity: intrinsic cytotoxicity of Ce6 and nanophotosensitizers was performed in the dark condition without irradiation of cells similarly as described in phototoxicity.

### 2.11. Intracellular Uptake of Nanophotosensitizers

HeLa cells (2 × 10^4^ cells/well) were seeded in 96-well plates and cultured overnight in 5% CO_2_ at 37 °C. These cells were treated with the Ce6 or FaPEGbCDseseCe6 nanophotosensitizers, as described above. 2 h later, the cells were washed twice with PBS and then solubilized in 50 µL of lysis buffer (GenDEPOT, Barker, TX, USA). These were used to measure the Ce6 uptake ratio using an Infinite M200 Pro microplate reader (Tecan) (excitation wavelength: 407 nm, emission wavelength: 664 nm).

### 2.12. Fluorescence Microscopy 

HeLa cells (3 × 10^5^) were seeded in 6-well plates with cover glass and then treated with either Ce6 or nanophotosenstitizers for 90 min. After that, the cells were washed twice with PBS and then fixed with 4% paraformaldehyde for 15 min at room temperature. The cells were washed with PBS twice and then immobilized with mounting solution (Immunomount, Thermo Electron Co., Pittsburgh, PA, USA). The cells were observed with a fluorescence microscope (Eclipse 80i; Nikon, Tokyo, Japan).

### 2.13. Flow Cytometry

HeLa cells (1 × 10^6^) in 6-well plates were treated with Ce6 or nanophotosensitizers (2 μg/mL Ce6 concentration) for 90 min. Thereafter, the cells were washed twice with PBS and then harvested via trypsinization. They were used for flow cytometry measurements (Invitrogen Attune NxT flow cytometers, ThermoFisher Scientific, Waltham, MA, USA).

### 2.14. ROS Generation Assay 

HeLa cells (2 × 10^4^ cells) seeded in a 96-well plate were cultured overnight in 5% CO_2_ at 37 °C. Thereafter, the cells were treated with either the Ce6 or nanophotosensitizers in phenol red-free media with the method that is described in the phototoxicity section. Intracellular ROS level was measured with a DCFH-DA assay as follows: DCFH-DA was added to the cell culture (final concentration of DCFH-DA: 20 µM) and incubated for 2 h at 37 °C [38]. Then, the cells were washed with PBS twice and replaced with fresh phenol red-free media (100 µL). The cells were irradiated at 664 nm (2.0 J/cm^2^). The intracellular ROS level was measured with a microplate reader (Infinite M200 pro microplate reader (Tecan Trading AG, Männedorf, Switzerland)). The excitation and emission wavelengths were 485 nm and 535 nm, respectively.

### 2.15. In Vivo Fluorescence Imaging and PDT Study

Fluorescence imaging: 1 × 10^6^ HeLa cells were subcutaneously administered into the backs of nude BAL b/C mice (male, 20 g, five weeks old). An aqueous nanophotosensitizer solution was sterilized with syringe filters (0.8 µm). When the solid tumor diameters became larger than 4 mm, nanophotosensitizer solution was intravenously (i.v.) administered through a tail vein (injection volume: 100 µL). 24 h later, the mice were sacrificed and observed with a Maestro^TM^ 2 small animal imaging instrument.

PDT treatment: 1 × 10^6^ HeLa cells were subcutaneously administered into the backs of nude BAL b/C mice (male, 20 g, five weeks old). When the solid tumor diameters became about 4 mm, the mice were divided into three groups: a control group, a Ce6 treatment group and a nanophotosensitizer treatment group. The injection dose of the Ce6 was adjusted to 10 mg/kg for each mouse. Each group had 5 mice. PBS was used for the control group. The Ce6 was dissolved into Cremophor EL^®^/ethanol (1/1) mixtures and then diluted with PBS (pH 7.4, 0.01 M) by ten times for injections. The nanophotosensitizers in deionized water were sterilized with syringe filters (0.8 µm) and diluted with PBS (pH 7.4, 0.01 M) in appropriate manners. Each treatment was administered intravenously via a tail vein of each mouse. Injection volume was 100 µL for the control and Ce6 treatment groups. For the nanophotosensitizer treatment group, the injection volume was 200 µL(52 mg nanophotosensitizers/kg; for a 20 g mouse, 1.04 mg nanophotosensitizers/0.2 mL PBS was used.). Two days later, the mice were anesthetized for PDT treatment (2.0 J/cm^2^, 664 nm). For irradiation of the mice, each mouse body was covered with fabric material to avoid interference of irradiated light except tumor mass. The day of the first irradiation was recorded as day 0 and, three days later, the mice were irradiated once more. The changes in tumor volume were measured with vernier calipers at 5-day intervals. The equation for the tumor volume: tumor volume (mm^3^) = (length × width^2^)/2.

All of the animal experiments in this study were carefully performed under the guidelines of the Pusan National University Institutional Animal Care and Use Committee (PNUIACUC). Furthermore, the protocol for this animal study was reviewed and monitored by the PNUIACUC in accordance with their ethical procedures and scientific care and was approved (approval number: PNU-2017-1610).

### 2.16. Statistical Analysis 

The statistical significance of the results was analyzed with Student’s test using SigmaPlot^®^ program.

## 3. Results

### 3.1. Synthesis of FaPEGbCDseseCe6 Conjugates

**Figure 1:**
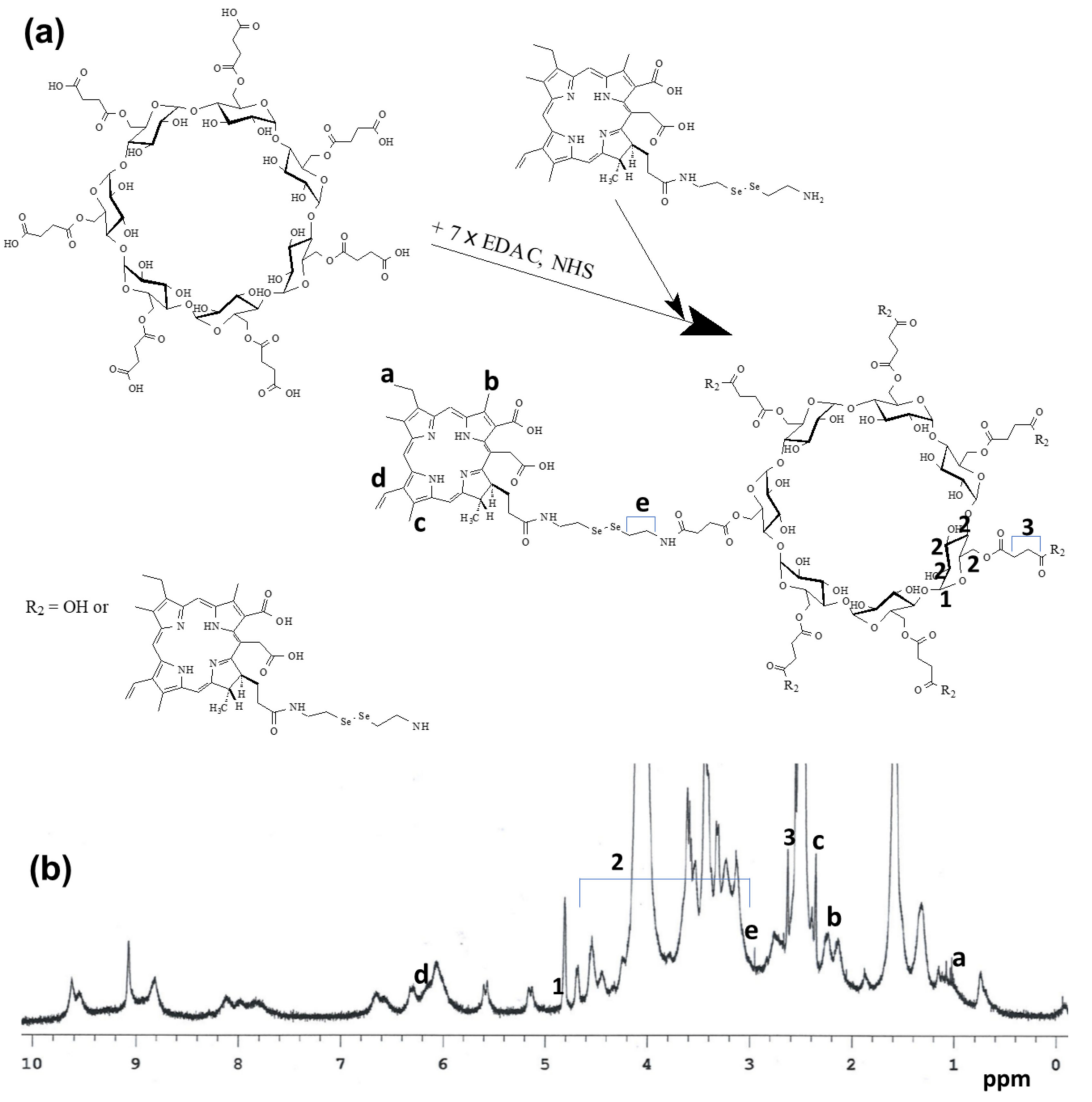
Synthesis scheme (**a**) and ^1^H NMR spectra (**b**) of succinyl β-cyclodextrin-selenocystamine-Ce6 conjugates. Succinyl β-cyclodextrin-selenocystamine-Ce6 conjugates was dissolved in dimethyl sulfoxide (DMSO)-d form to measure the chemical structure with ^1^H NMR spectroscopy.

To synthesize the FaPEGbCDseseCe6 conjugates, the Ce6 was conjugated with selenocystamine and then these conjugates were conjugated again with succinyl β-cyclodextrin to produce succinyl β-cyclodextrin-selenocystamine-Ce6 conjugates, as shown in Figure S1 and Figure 1. One of the carboxyl groups of the Ce6 was activated with the EDAC/NHS system and then conjugated with an amine group of selenocystamine (Ce6-selenocystamine conjugates). As shown in Figure S1, the ^1^H NMR spectra showed that specific peaks of the Ce6 were confirmed at 1~8 ppm, while those of ethylene protons of selenocystamine were confirmed at 3.0~3.2 ppm. The Ce6-selenocystamine conjugates showed specific peaks from both the Ce6 and selenocystamine, as shown in Figure S1. This conjugate was attached to the succinyl β-cyclodextrin, as shown in Figure 1. The carboxyl group of succinyl β-cyclodextrin was activated with the EDAC/NHS system and then conjugated with Ce6-selenocystamine. The unreacted by-products were removed using a dialysis procedure. The yield of the succinyl β-cyclodextrin-selenocystamine-Ce6 conjugates was higher than 92% *(w/w*). The number of peaks of succinyl β-cyclodextrin was confirmed to be between 2 and 7, as shown in Figure S2. Figure 1 shows the peaks of each component of succinyl β-cyclodextrin-selenocystamine-Ce6 conjugates, which are succinyl β-cyclodextrin, selenocystamine and Ce6. These results indicated that the Ce6-selenocystamine conjugates were successfully conjugated with succinyl β-cyclodextrin, which produced succinyl β-cyclodextrin-selenocystamine-Ce6 conjugates.

**Figure 2:**
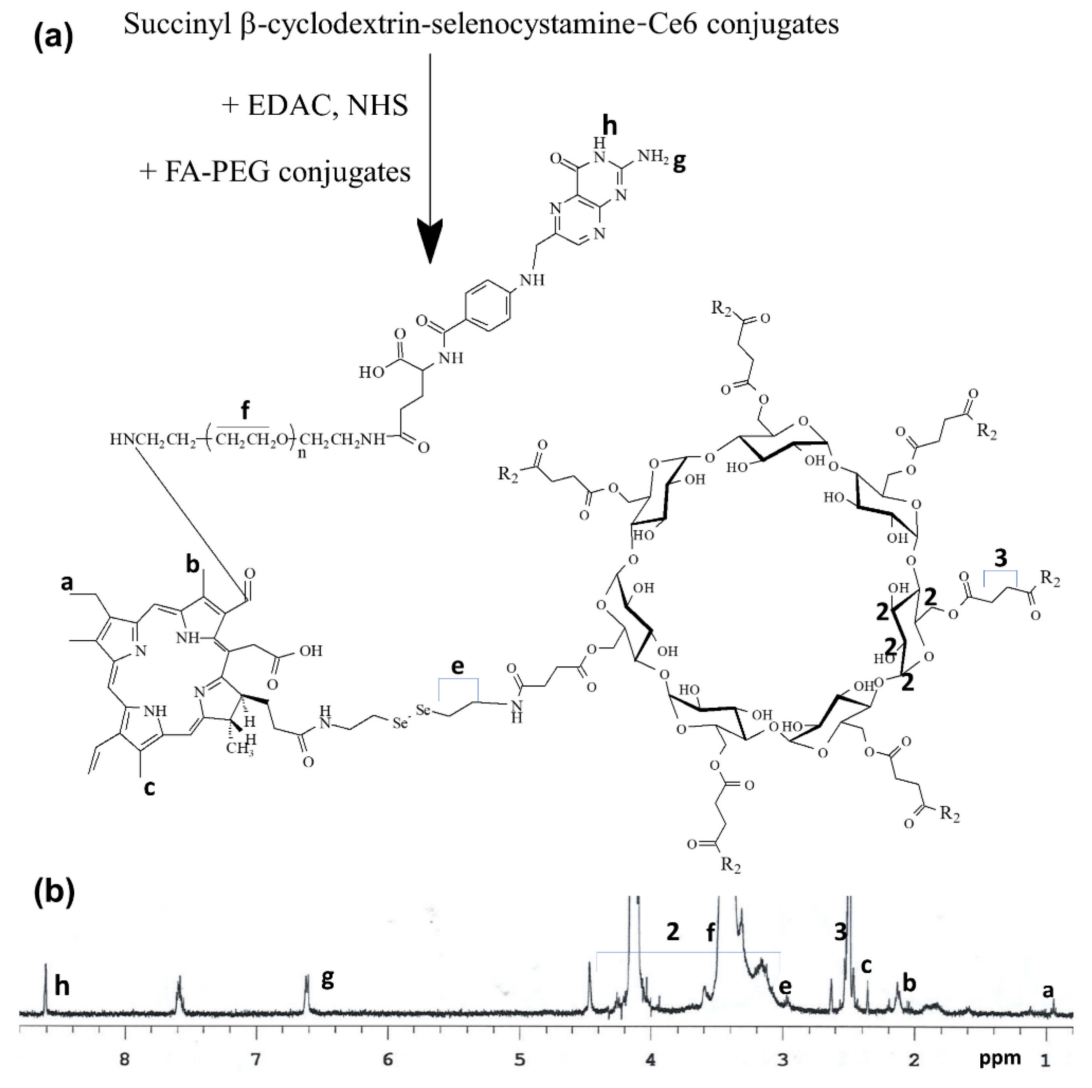
Synthesis scheme (**a**) and ^1^H NMR spectra (**b**) of FaPEGbCDseseCe6 conjugates.

To synthesize the succinyl β-cyclodextrin-selenocystamine-Ce6 conjugates, FA-PEG was synthesized, as shown in Figure S3. The carboxyl group of FA was conjugated with one of the amine groups of PEG, which then produced FA-PEG conjugates. The peaks of FA were confirmed to be between 1 and 9 ppm, while the peaks of the ethylene protons of PEG were confirmed at 3.4~3.7 ppm, as shown in Figure S3. FA-PEG conjugates were conjugated with succinyl β-cyclodextrin-selenocystamine-Ce6 conjugates to produce the FaPEGbCDseseCe6 conjugates, as shown in Figure 2. The yield of the FaPEGbCDseseCe6 conjugates was higher than 94% (*w/w*). The peaks of the FaPEGbCDseseCe6 conjugates were also confirmed, as shown in Figure 2. The peaks of each component, such as Ce6, selenocystamine, PEG, FA and succinyl β-cyclodextrin, were confirmed using ^1^H NMR spectra. These results indicate that the FaPEGbCDseseCe6 conjugates were successfully synthesized.

### 3.2. Fabrication and Characterization of Nanophotosensitizers of FaPEGbCDseseCe6 Conjugates

**Figure 3:**
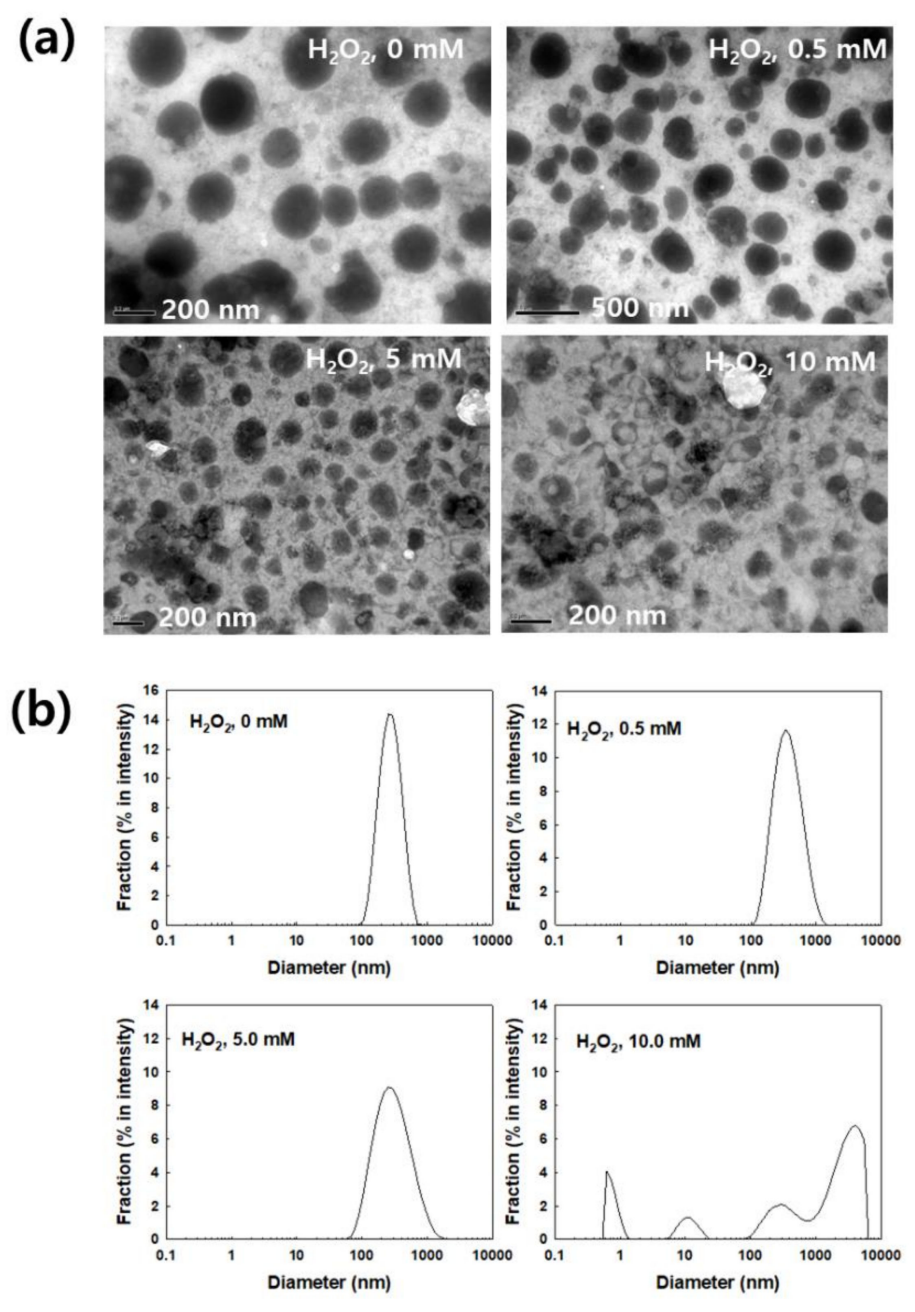
(**a**) The effect of H_2_O_2_ on the morphological changes in the nanophotosensitizers of the FaPEGbCDseseCe6 conjugates. (**b**) The effect of H_2_O_2_ on the changes of particle size distribution of nanophotosensitizers.

**Figure 4:**
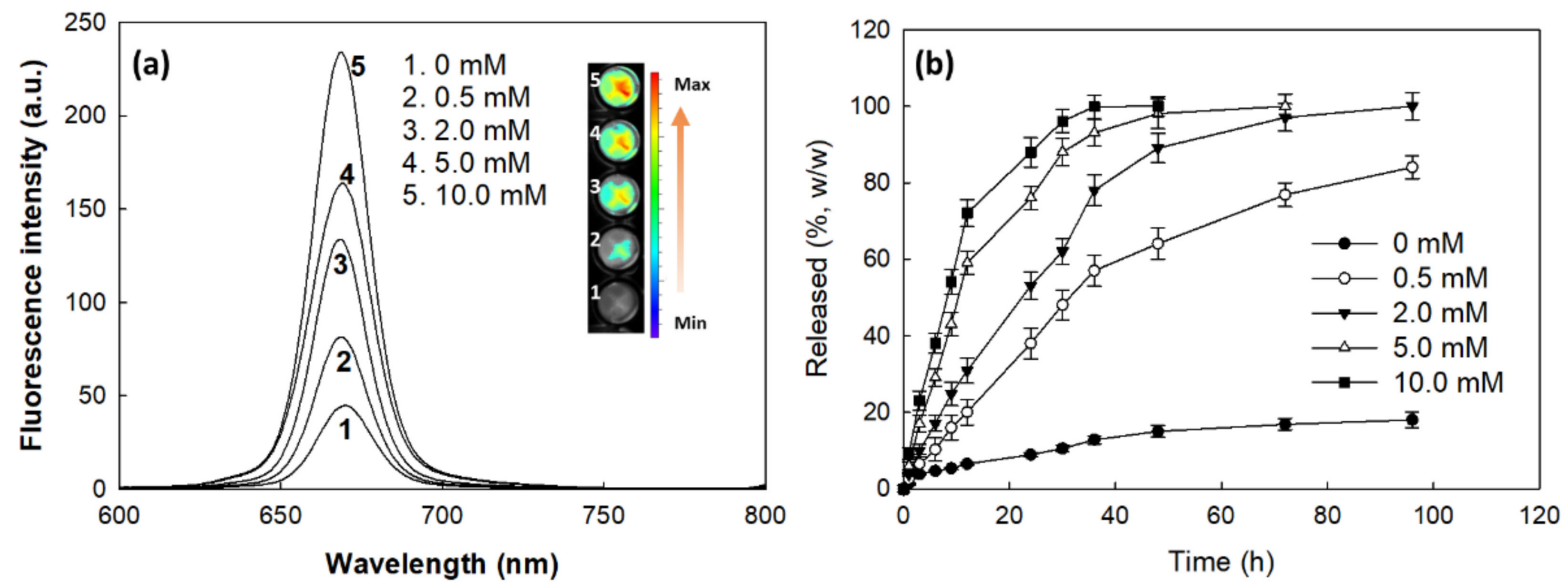
(**a**) The effect of H_2_O_2_ on the changes in the fluorescence spectra of the nanophotosensitizers of the FaPEGbCDseseCe6 conjugates. (**b**) The effect of H_2_O_2_ on the Ce6 release from the nanophotosensitizers. The Ce6 concentration was adjusted to 0.1 mg/mL in PBS with or without H_2_O_2_. This solution was incubated for 4 h at 37 °C to be reacted with H_2_O_2_.

To measure the content of Ce6 in the FaPEGbCDseseCe6 conjugates, the FaPEGbCD-seseCe6 conjugates were distributed into the PBS in the H_2_O_2_. The measured Ce6 content was approximately 19.2% (*w/w*). To fabricate the FaPEGbCDseseCe6 conjugates, they were dissolved in DMSO/water mixtures and then dialyzed against water. These were used to analyze the physicochemical properties and ROS sensitivity of the nanophotosensitizers, as shown in Figure 3 and Figure 4.

To test the ROS sensitivity, the nanophotosensitizers of FaPEGbCDseseCe6 conjugates were incubated in the PBS solution in various concentrations of H_2_O_2_. Then, the changes of morphology of the nanophotosensitizers were observed, as shown in Figure 3a. The nanophotosensitizers of FaPEGbCDseseCe6 conjugates showed spherical shapes, as shown in Figure 3a. Their average sizes were less than 300 nm. When H_2_O_2_ was added to the aqueous solution of nanophotosensitizers, they began to morphologically disintegrate more with an increased concentration of H_2_O_2_. This means that nanophotosensitizers of FaPEGbCDseseCe6 conjugates maintained spherical morphologies in the absence of H_2_O_2_ and, in the presence of 0.5 mM H_2_O_2_, some of them started to be crushed or disintegrated. Then, the disintegrated form of nanophotosensitizers was increased in the higher H_2_O_2_ concentrations and, then the most of the nanophotosensitizers disintegrated in the 10 mM H_2_O_2_ concentration. The particle size distribution of nanophotosensitizers also supported these results, as shown in Figure 3b; i.e., they maintained narrow and monomodal distribution patterns in the absence of H_2_O_2_. Their average diameter was 248.3 ± 24 nm in the absence of H_2_O_2_. However, the average particle size and size distribution became bigger and wider, respectively. Furthermore, the nanophotosensitizers gained a multimodal size distribution in the 10 mM H_2_O_2_ concentration, as shown in Figure 3b. These results mean that the nanophotosensitizers must have been swollen by H_2_O_2_ and, after that, they disintegrated at a high concentration of H_2_O_2_. These results indicated that nanophotosensitizers of the FaPEGbCDseseCe6 conjugates displayed ROS sensitivity and their physicochemical properties could be altered in the presence of ROS.

Figure 4 shows the fluorescence intensity and Ce6 release behavior of the nanophotosensitizers of the FaPEGbCDseseCe6 conjugates. As shown in Figure 4a, the fluorescence intensity gradually increased with the increase of H_2_O_2_ concentration, indicating that liberation of Ce6 from nanophotosensitizers was accelerated and dependent on the H_2_O_2_ concentration while Ce6 release from nanophotosensitizers was very slow in the absence of H_2_O_2_. These results indicate that nanophotosensitizers of FaPEGbCDseseCe6 conjugates have ROS-sensitivity.

### 3.3. Cell Culture and PDT Study In Vitro

**Figure 5:**
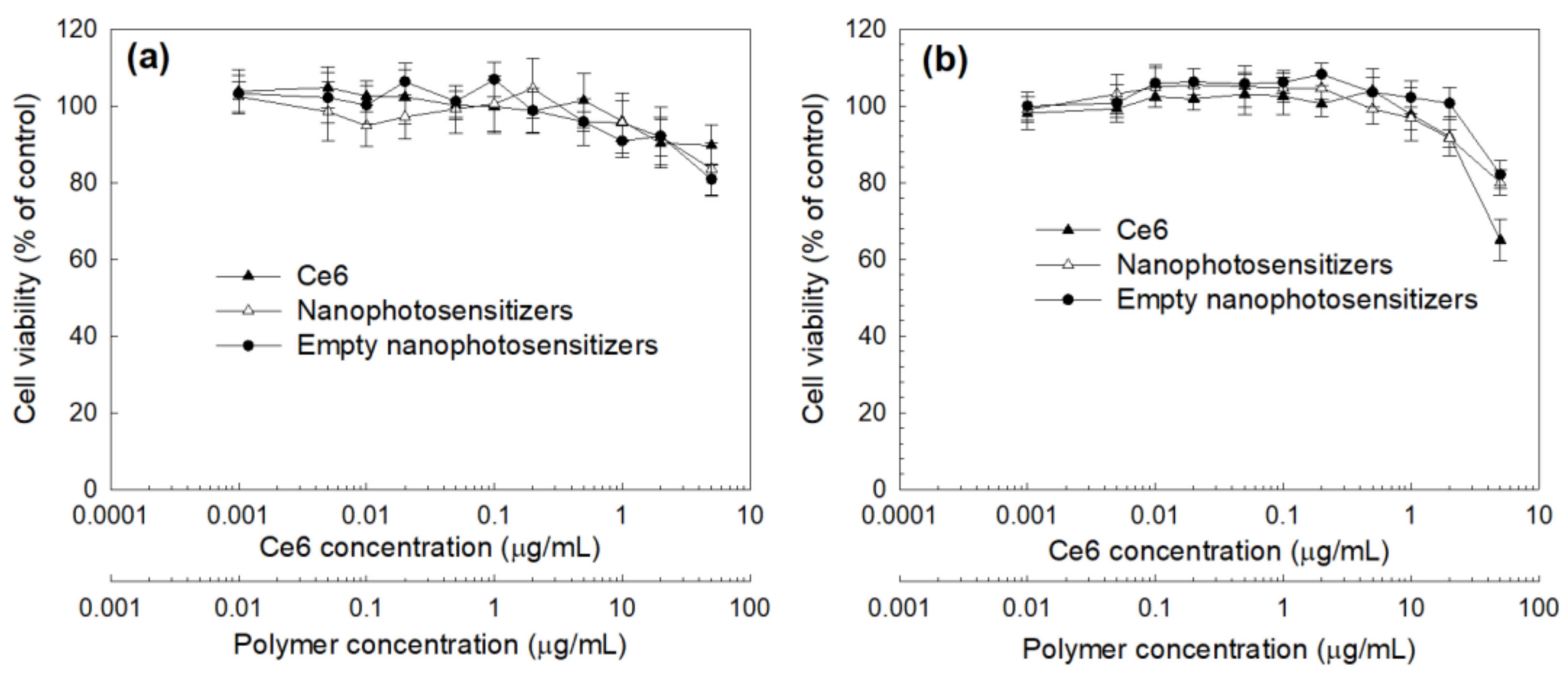
Dark toxicity of the Ce6 alone, nanophotosensitizers and empty nanophotosensitizers against CCD986SK human skin fibroblast cells (**a**) and HeLa human cervical carcinoma cells (**b**).

CCD986Sk human skin fibroblast cells and HeLa human cervical cancer cells were used to assess dark toxicity and PDT efficacy of nanophotosensitizers of FaPEGbCDseseCe6 conjugates. As shown in Figure 5a, Ce6 alone has no significant cytotoxicity against CCD986Sk cells in the absence of light. Also, nanophotosensitizers did not reveal intrinsic cytotoxicity until 5 μg/mL Ce6 concentration. These results mean that both of the Ce6 alone and nanophotosensitizers were not significantly toxic to normal cells (CCD986Sk) and cancer cells (HeLa) less than 5 μg/mL Ce6 concentration in the absence light irradiation since cell viability was maintained higher than 80% at these concentration range. Empty nanophotosensitizers were not significantly toxic until 50 μg/mL polymer concentration. Otherwise, the Ce6 alone has small dark-toxicity against HeLa cells as shown in Figure 5b; i.e., cell viability was decreased less than 70% when Ce6 concentration was 5 μg/mL. However, nanophotosensitizers maintained higher than 80% cell viability. Furthermore, empty nanophotosensitizers also maintained higher than 80% cell viability until 50 μg/mL of the polymer concentration. These results indicate that both of the Ce6 alone and nanophotosensitizers have no dark toxicity against normal cells less than 5 μg/mL Ce6 concentration.

**Figure 6:**
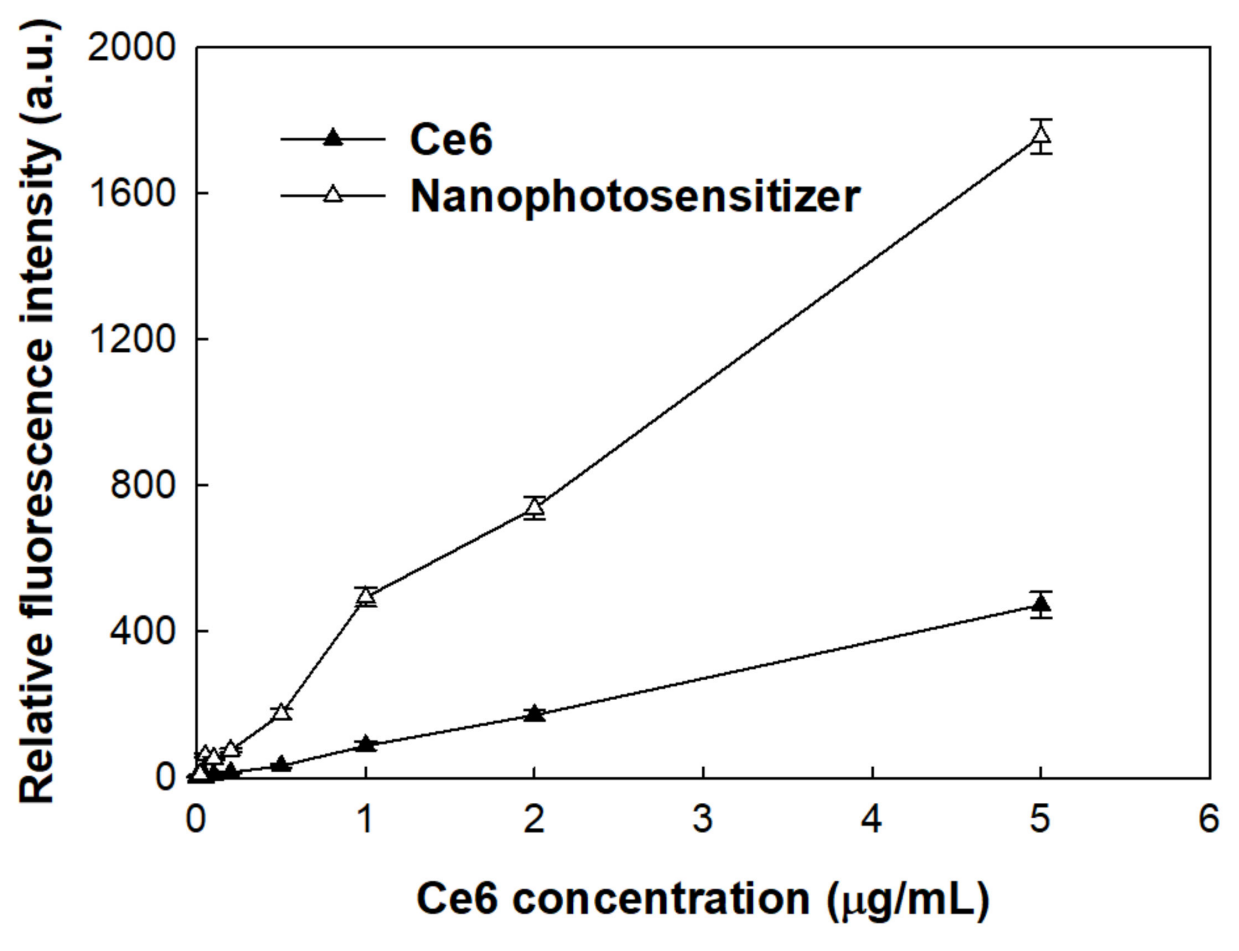
Ce6 uptake by HeLa cells. Cells were treated with Ce6 or FaPEGbCDseseCe6 nanophotosensitizers for 2 h and then measured fluorescence intensity as a Ce6 uptake ratio (Excitation wavelength of 407 nm, emission wavelength of 664 nm).

**Figure 7:**
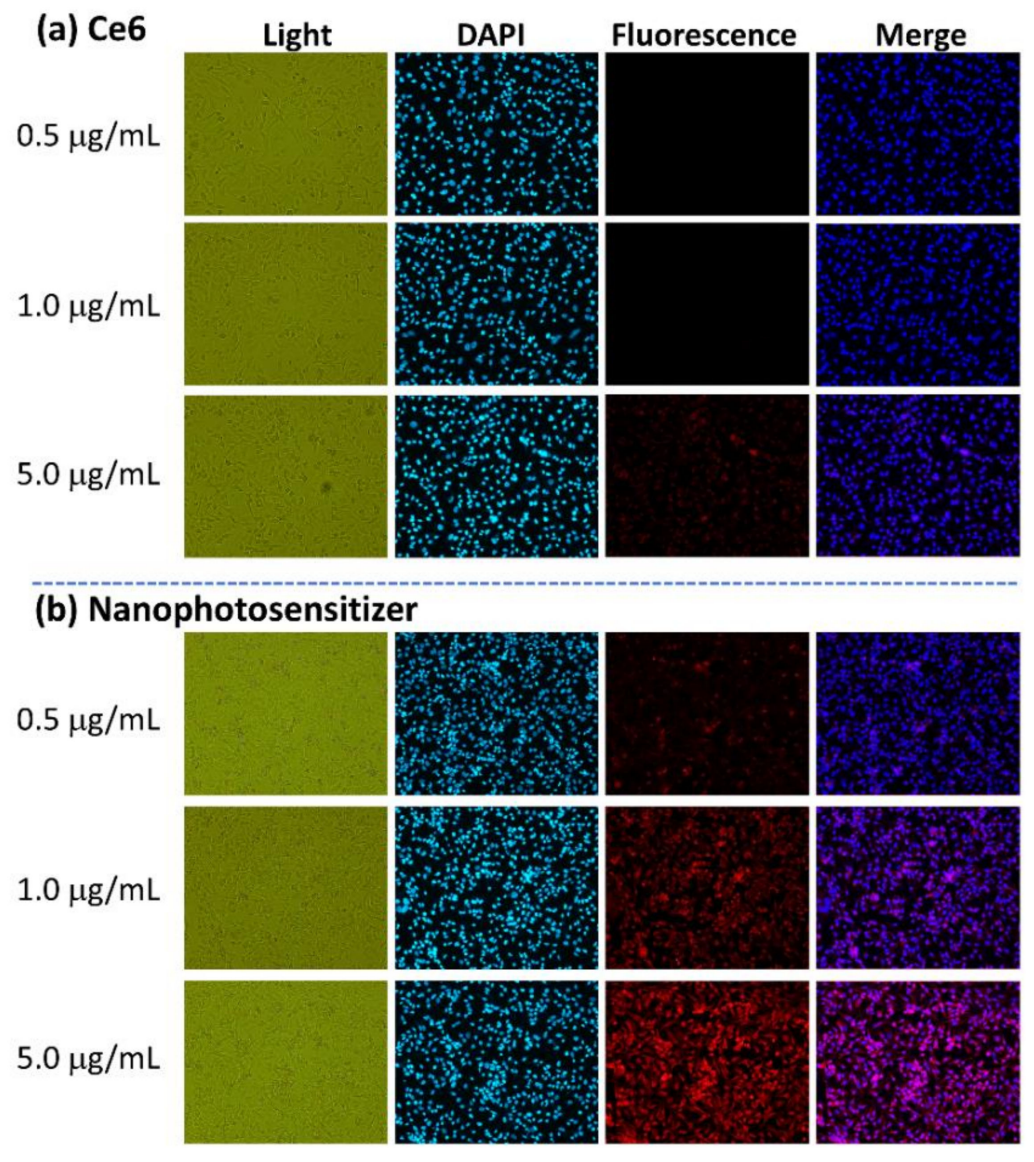
Fluorescence observation of the HeLa cells with treatment using the Ce6 (**a**) and the nanophotosensitizers (**b**). Treatment was similar to Figure 6. Magnification: ×100.

Figure 6 and Figure 7 show the Ce6 uptake by HeLa cells. As shown in Figure 6, the Ce6 uptake ratio by HeLa cells gradually increased according to the increase of Ce6 concentration both of the Ce6 alone and the nanophotosensitizers. The Ce6 uptake ratio in the treatment of nanophotosensitizers was significantly higher than that of the Ce6 alone. Nanophotosensitizers showed approximately 3.8 times higher Ce6 uptake ratio than that of Ce6 alone. Figure 7 supports these results, i.e., the observation of HeLa cells using fluorescence microscopy indicated that red fluorescence, which represents the Ce6 uptake, increased according to the increase of Ce6 concentration. Especially, the red fluorescence intensity in the nanophotosensitizer treatment was significantly higher than that in the treatment of Ce6 alone. These results indicate that nanophotosensitizers have great capability for intracellular uptake compared to Ce6 alone.

**Figure 8:**
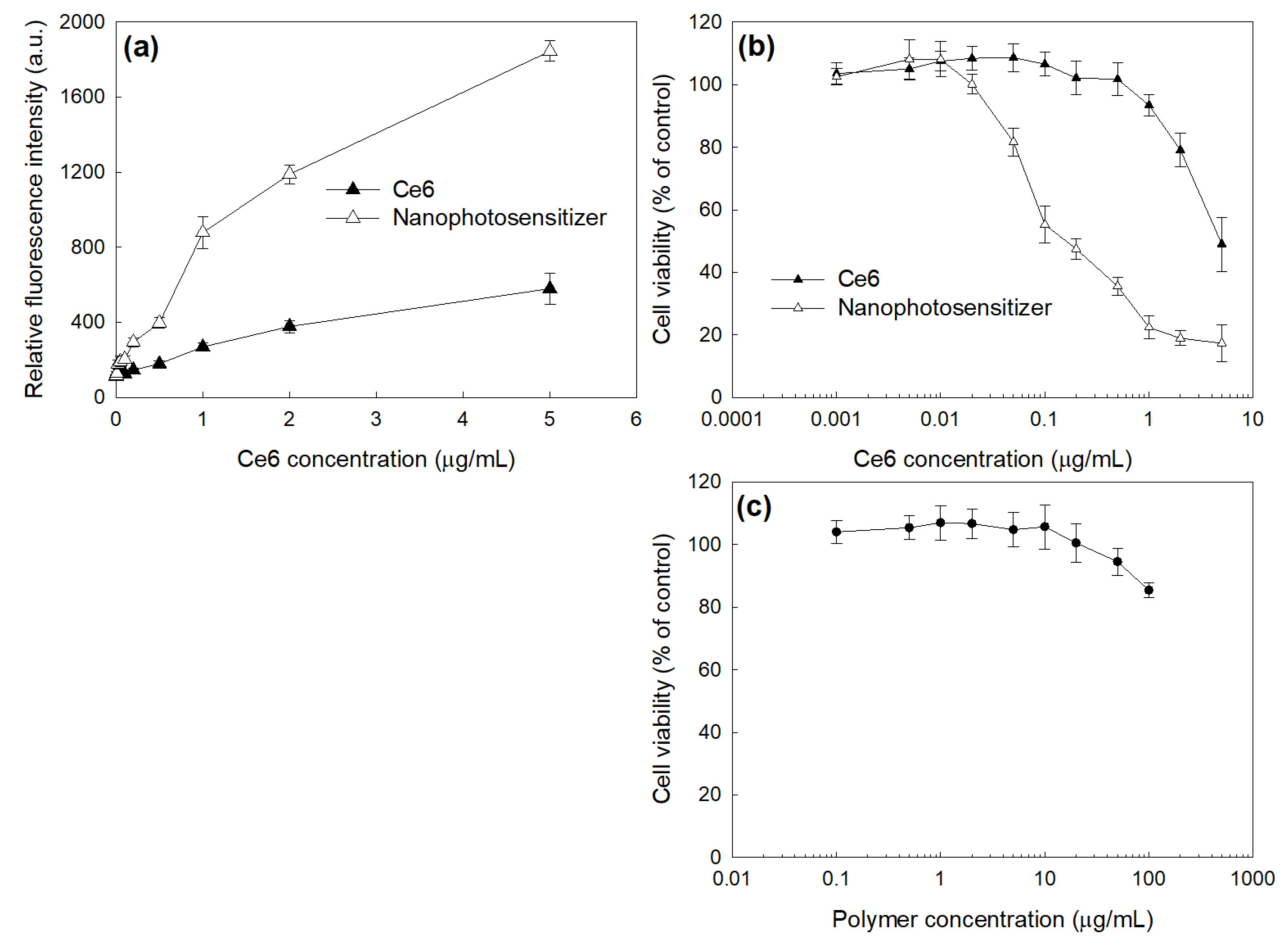
The effect of the Ce6 alone or nanophotosensitizers on the ROS generation (**a**) and phototoxicity (**b**) against HeLa cells. (**c**) The phototoxicity of empty nanophotosensitizers. The ROS generation was measured with a DCFH-DA assay. Cells (2 × 10^4^ cells/well in 96 wells) were irradiated at 664 nm (2 J/cm^2^). All values are average ± S.D. from results of a single independent experiment with eight replicates.

To assess the PDT efficacy, the HeLa cells treated with the Ce6 alone or the nanophotosensitizers were irradiated at 664 nm. Figure 8 shows the ROS generation (Figure 8a) and phototoxicity (Figure 8b) of the Ce6 alone and nanophotosensitizers against HeLa cells. As expected, the ROS generation by the treatment of nanophotosensitizers was significantly higher than that of the Ce6 alone. On the contrary, the intracellular ROS generation in HeLa cells was negligible in the absence of light irradiation for both the Ce6 alone and nanophotosensitizers (Figure S6). Furthermore, the phototoxicity in the treatment of the nanophotosensitizers was also significantly higher than that of the Ce6 alone, i.e., the cell viability after the nanophotosensitizer treatment was less than 30%, while that of the Ce6 alone was higher than 80%. Altogether, these results indicated that the nanophotosensitizers showed an improved intracellular uptake ratio, superior ROS generation and definite phototoxicity.

**Figure 9:**
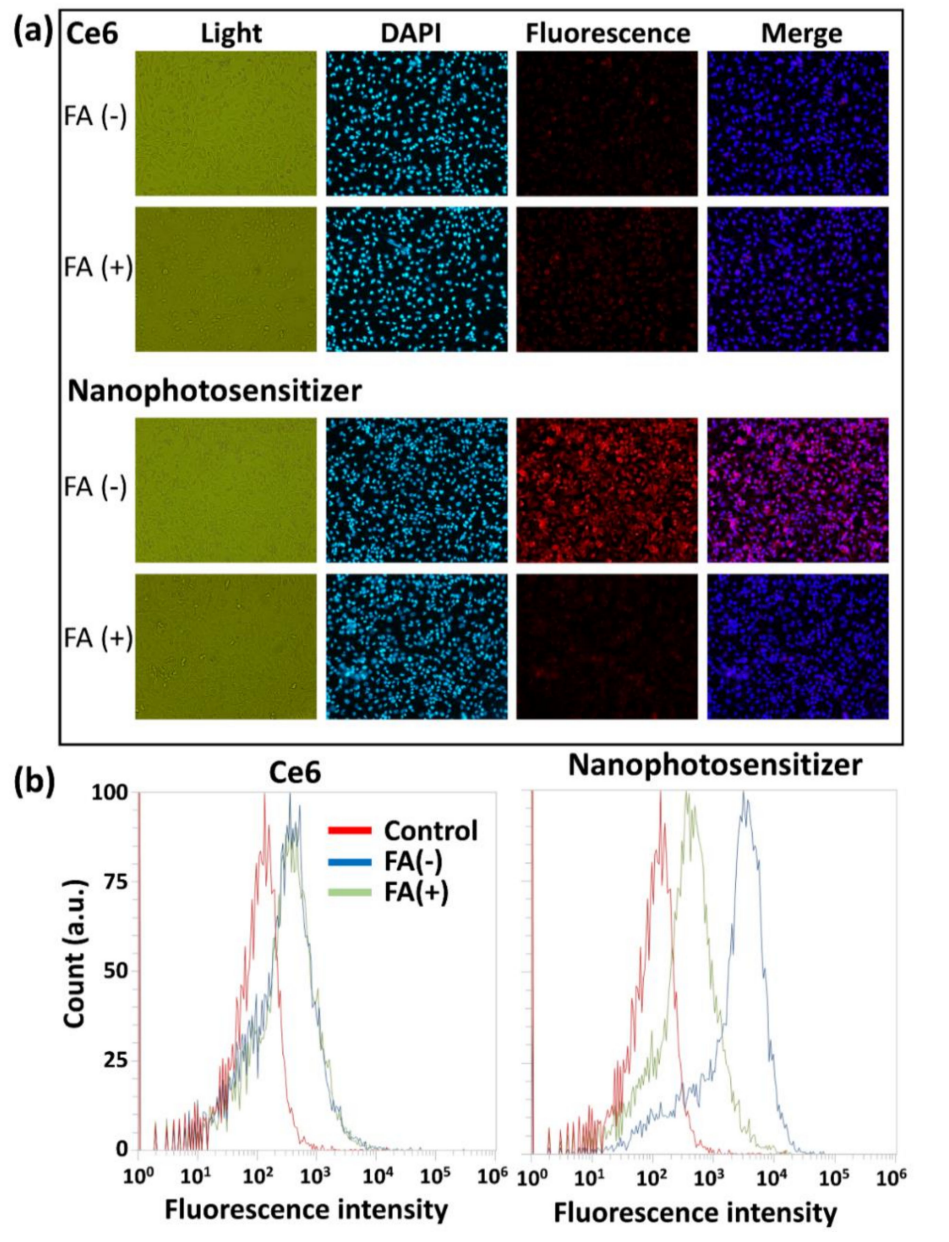
Fluorescence observations (**a**) and flow cytometry results (**b**) of HeLa cells. The folate receptors of cancer cells were blocked with a pretreatment of FA (10 mM) 30 min before a treatment of Ce6 alone or nanophotosensitizers. For the flow cytometry measurement, cells (1 × 10^6^ cells/well in 6 wells) were treated with the Ce6 alone or nanophotosensitizers (2 μg/mL Ce6 concentration) for 90 min and then washed with PBS. Magnification: ×100.

**Figure 10:**
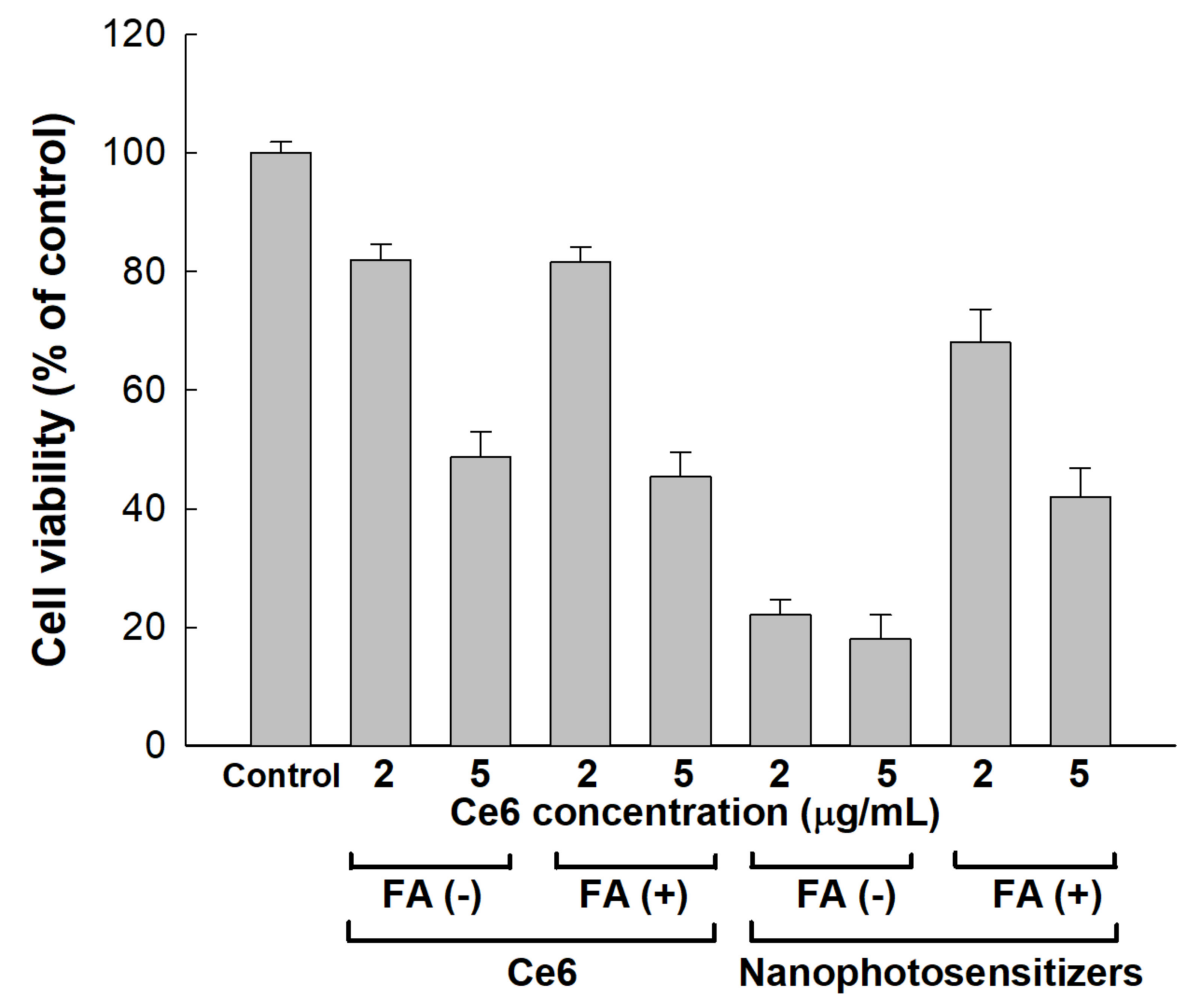
The effect of folate receptor blocking on the viability of HeLa cells. Cells (2 × 10^4^ cells/well in 96 wells) were treated with Ce6 or nanophotosensitizers, as described in Figure 9. For blocking the folate receptors, FA (10 mM) was used to pretreat the cells for 30 min to block the folate receptors. Then, the cells were washed with PBS, treated with the Ce6 alone or nanophotosensitizers (2 or 5 μg/mL Ce6 concentration) for 2 h and irradiated at 664 nm (2 J/cm^2^).

Since nanophotosensitizers of FaPEGbCDseseCe6 conjugates have folic acid at the end of PEG domain, their targeting capacity against the folate receptors of HeLa cells were evaluated by blocking folate receptor of cells as shown in Figure 9 and Figure 10. The excess amount of folic acid was primarily used to treat the cells to block the folate receptors of HeLa cells and, thereafter, the Ce6 or nanophotosensitizers were treated. As shown in Figure 9, blocking the folate receptor did not affect the fluorescence intensity in the HeLa cells when the Ce6 treatment was used. However, the treatment with nanophotosensitizers resulted in decrease of red fluorescence intensity by blocking the folate receptor (FA(+)), while a strong red fluorescence intensity was observed in the absence of folate receptor blocking (FA(−)) (Figure 9a and S5). Furthermore, the flow cytometry also supported these results, as shown in Figure S4 and Figure 9b. In Ce6 treatment, fluorescence intensity was not changed with or without blocking of folate receptor. However, the fluorescence intensity in treatment of nanophotosensitizers significantly changed by blocking the folate receptor (FA(+)), as shown in Figure 9b. These results indicated that nanophotosensitizers of FaPEGbCDseseCe6 conjugates were specifically delivered to HeLa cells via a folate receptor-mediated manner. Figure 10 shows the changes in phototoxicity of nanophotosensitizers via folate receptor blocking. The nanophotosensitizers showed higher phototoxicity than that of the Ce6 alone. In the Ce6 treatment, the blocking of the folate receptor did not significantly change cell viability as shown in Figure 10. However, the cell viability was significantly changed in nanophotosensitizer treatment by blocking the folate receptor, i.e., the cell viability was less than 30% without blocking of folate receptor while the cell viability was increased to higher than 40% at 5 μg/mL and higher than 60% at 2 μg/mL Ce6 concentrations. These results indicate that nanophotosensitizers of FaPEGbCDseseCe6 conjugates can be specifically delivered to HeLa cells via folate receptor-mediated pathway.

### 3.4. In Vivo Animal Tumoxenograft Study Using HeLa Cells

**Figure 11:**
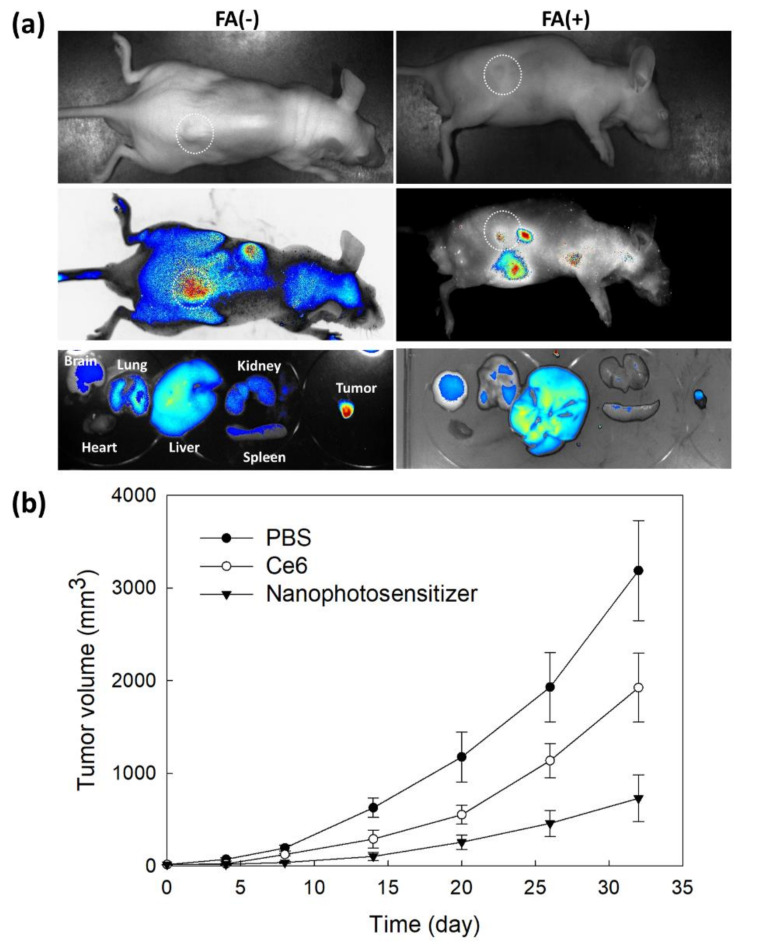
(**a**) In vivo animal imaging using a tumor xenograft model of HeLa cells. Fluorescence imaging of a HeLa-cell-derived tumor xenograft model of mice. Prior to the injection of the Ce6 or nanophotosensitizers, FA in PBS (pH 7.4, 0.01 M, 20 mg/kg) was i.v. injected to mice via tail veins. After that, the Ce6 or nanophotosensitizers (10 mg/kg as the Ce6 concentration) were i.v. administered. Twenty-four hours later, the mice were sacrificed and then observed for fluorescence images using Maestro II^®^. (**b**) The effect of PDT on the growth of tumor masses. The Ce6 or nanophotosensitizers (10 mg/kg as a Ce6 concentration) were i.v. administered. Two days later, the mice were anesthetized for PDT treatment (2.0 J/cm^2^, 664nm). The day of the first irradiation was recorded as day 0 and, three days later, the mice were irradiated once more. Equation of tumor volume: tumor volume (mm^3^) = (length × width^2^)/2.

To evaluate the biodistribution and PDT efficacy of the nanophotosensitizers, HeLa cells were administered to the backs of mice to generate tumor xenografts. To assess the folate receptor targetability of the nanophotosensitizers, folic acid was intravenously (i.v.) administered via tail veins of the mice prior to injection of the nanophotosensitizers. As shown in Figure 11a, the fluorescence intensity in the absence of folate receptor blocking (FA(−)) was stronger than that of other organs. However, the fluorescence intensity was relatively lower than that of other organs when the folate receptors were blocked (FA(+)). These results indicated that the nanophotosensitizers of FaPEGbCDseseCe6 conjugates could be delivered to HeLa tumors through a folate-receptor-mediated pathway in vivo. To assess the PDT efficacy, nanophotosensitizers were i.v. administered and then mice were irradiated two times. As shown in Figure 11b, the tumor volume gradually increased. Even though both the Ce6 and nanophotosensitizers properly inhibited the growth of tumor mass, the nanophotosensitizers had higher efficacy in inhibition of the growth of tumor masses than that of the Ce6 alone. The in vivo animal study using the HeLa tumor xenograft model also showed that the nanophotosensitizers of FaPEGbCDseseCe6 conjugates had superior delivery capacity against HeLa tumors and then they properly inhibited the growth of the tumor masses.

## 4. Discussion

The physiological state of a tumor microenvironment is quite different compared to normal tissues [39,40,41]. A tumor microenvironment is characterized by abundant intra/extracellular enzymes, over-expression of various molecular receptors, acidic pH, abnormal redox potential and low temperature [39,40,41]. In particular, the redox potential is normally elevated in the tumor microenvironment [42,43,44,45]. For example, oxidative stress in a tumor microenvironment is generally higher than that of normal tissues and level of glutathione (GSH), a typical antioxidant molecule, is also untampered in tumor microenvironment [42,43,44,45]. This abnormal redox status is deeply related to tumor progression, metastasis and angiogenesis [45,46]. Paradoxically, an abnormal ROS level is frequently considered as a cancer-targeting strategy [47]. Even though appropriate ROS level in a tumor microenvironment is regarded as one of the major reasons for the promotion of progression and metastasis of cancer, where a higher ROS level in cancer cells than that for the survival of cancer cells induces apoptosis and/or necrosis of cancer cells [48,49]. For example, piperlongumine increases intracellular ROS accumulation and then depletes GSH in cancer cells [48]. This status induces a decrease in the proliferation and migration of breast cancer cells [48]. Furthermore, Lee et al. reported that redox-sensitive nanoparticles amplify the anticancer activity of piperlongumine [49]. In particular, diselenide linkage can be disconnected by ROS in cancer cells [49,50]. Wei et al. reported that diselenide-based polymers accumulate an excessive level of intracellular ROS and then induce apoptosis in cancer cells [50]. Our results (Figure 3 and Figure 4) showed that H_2_O_2_, which is a typical ROS, induced the disintegration of the nanophotosensitizers and then accelerated Ce6 release from the nanophotosensitizers of FaPEGbCDseseCe6 conjugates. Furthermore, these phenomena reflect that intracellular disintegration of the nanophotosensitizers can be accelerated through ROS generation in the cells. Then, the nanophotosensitizer-based PDT of HeLa cells might be amplified through this process of oxidative stress (ROS generation to nanophotosensitizer disintegration to ROS generation). Furthermore, these processes accelerate the death of HeLa cells (Figure 8). Lee et al., also reported that chitosan nanoparticles having diselenide linkages were seriously disintegrated by addition of 10 mM or 20 mM H_2_O_2_ and then the release rate of anticancer agent was accelerated [49]. The ROS-sensitive disintegration of nanoparticles is regarded as a promising concept for intracellular delivery of anticancer drugs.

Reginato et al.’s review showed that oxidative stress that is mediated by PDT contributes to distorting the redox-signaling pathways in cancer cells, resulting in changes in death signals, abnormal proliferation and repair process [51]. This status may induce inflammatory cytokines, abnormal molecular signals and multiple stresses. These events also induce an inflammatory stress-associated immune response and modified cross-talk between neighboring/distant cells and inflammatory-injured cells [52]. Furthermore, oxidative stress in cancer cells, such as via Ce6-based PDT, may induce a defensive mechanism in cancer cells and then activate anti-oxidants pathways [38]. PDT efficacy can be significantly affected by the total glutathione levels and the glutathione peroxidase and glutathione reductase activities. The defensive process against PDT-mediated oxidative stress is also associated with photosensitizer-resistant behavior via antioxidant detoxifying enzymes and the activation of heat-shock proteins [53]. In consideration of these events, nano-dimensional carriers, such as nanophotosensitizers, can be considered as one of the promising solutions [31,32,33,34,35,36,37].

**Figure 12:**
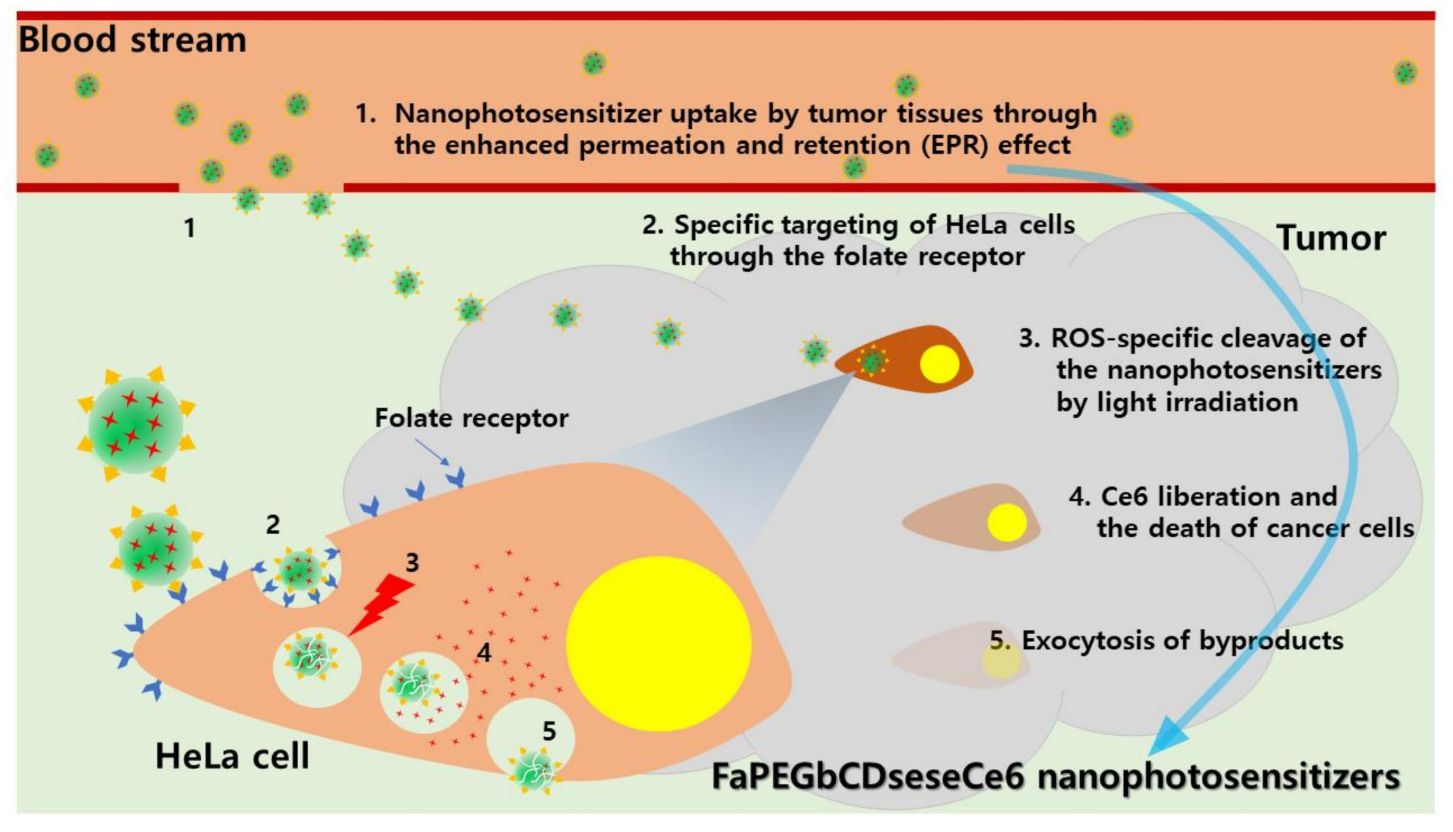
Schematic illustrations of tumor targeting of FaPEGbCDseseCe6 nanophotosensitizers and the mechanism of improved PDT.

Nano-dimensional carriers composed of high molecular weight molecules such as polysaccharide, protein and synthetic polymers have distinct properties compared to traditional small molecules and anticancer agents in vivo [39,40,41]. Since the research group of Prof. Maeda demonstrated the enhanced permeation and retention (EPR) effect of macromolecules and macromolecular-based drug carriers in tumor tissues, various drug carriers such as nanoparticles, polymeric micelles, polymeric prodrugs, liposomes and protein-based drug carriers have been extensively investigated for passive or active tumor targeting [19,21,22,31,32,33,34,54,55,56,57]. Even though targeting moiety such as folic acid induces favorable accumulation of nanocarriers in the tumor cells, EPR effect is primarily considered as a dominant delivery mechanism when nanocarriers are administered systemically and, after that, they can target individual cancer cells as shown in Figure 12 [57]. Various molecular receptors are abundantly expressed in cancer cells compared to their normal counterparts. Among them, folate receptors are normally overexpressed in various types of cancer [58]. For example, folate receptor β, which is expressed on myeloid lineage hematopoietic cells, is highly expressed on malignant blasts in patients having myeloid leukemia and tumor-associated macrophages in the tumor microenvironment [59]. These properties of folate receptor enable nanocarriers to target cancer cells specifically and allow for application of nanocarriers in direct/indirect therapy of cancer. Furthermore, folate receptors are known to associate with the carcinogenesis of cervical cancers and they regulate the growth of cervical cancer cells [58]. Scientists reported that the downregulation of folate receptors, which are overexpressed in cervical cancer, resulted in the apoptosis and suppression of cervical cancer cells [58,60]. Interestingly, folate receptors can be used to detect cervical cancer cells since they typically overexpress folate receptors [61]. Lee et al. reported that FA-decorated nanoparticles increased the folate-receptor-specific delivery of anticancer drugs against KB human squamous carcinoma cells [62]. They argued that FA-decorated nanoparticles improved intracellular doxorubicin delivery via a folate-receptor-mediated pathway, i.e., the fluorescence intensity decreased when folate receptors were blocked and then intracellular delivery of doxorubicin decreased. Our results also showed that the nanophotosensitizers of FaPEGbCDseseCe6 conjugates were efficiently delivered into HeLa tumor cells in vitro and in vivo via the folate receptors of HeLa cells. Blocking folate receptors also regulated the PDT efficacy of the nanophotosensitizers (Figure 10 and Figure 11). In particular, folate receptor blocking by the FA pre-treatment significantly lowered the delivery capacity of the nanophotosensitizers in the tumor xenograft, indicating that the unwanted delivery of the nanophotosensitizers can be minimized against their normal counterpart and then the PDT efficacy against cervical cancer can be maximized through folate-receptor-specific delivery of photosensitizers. As depicted in Figure 12, nanophotosensitizers can be delivered to tumors via the enhanced permeation and retention (EPR) effect of tumors, internalized into tumor cells via specific interactions with folate receptors, disintegrated in the intracellular compartment by ROS generation and then kill the tumor cells. Practically, the nanophotosensitizers resulted in superior PDT efficacy against the HeLa tumor xenograft model compared to the Ce6 alone.

Moreover, the intracellular fate of photosensitizers and/or nanophotosensitizers also greatly affects the PDT efficacy against cancer cells [63,64]. Tsubone et al. reported that nanocarriers improve the yield of singlet oxygen generation in cells and delivery potential against mitochondria/lysosomes [63]. They argued that liberated photosensitizers from nanocarriers in the intracellular organelles have advantages for targeting intracellular organelles, such as mitochondria and lysosomes, and enhancing PDT-induced cell death. Martins et al. also reported that PDT-mediated cell death can be significantly improved by parallel damage in the membranes of mitochondria and lysosomes through autophagy malfunction [64]. Therefore, nanophotosensitizers have advantages in the production of intracellular ROS and then boost the damage of intracellular organelles, such as mitochondria or lysosomes. We expect that the enhanced intracellular delivery of nanophotosensitizers with cancer-cell-specific targeting may greatly improve the PDT efficacy of traditional photosensitizers. In the present study, the PDT efficacy and phenomenon of nanophotosensitizers of FaPEGbCDseseCe6 conjugates were still limited in vitro cellular cytotoxicity and in vivo anticancer activity. Compared to traditional photosensitizers such as Ce6, intracellular reactions based on mitochondria and lysosomes must be differently appeared by the treatment with nanophotosensitizers of FaPEGbCDseseCe6 conjugates. We will also investigate the intracellular targeting of nanophotosensitizers regarding mitochondria/lysosomes in future research.

## 5. Conclusions

FaPEGbCDseseCe6 conjugates were synthesized for ROS-enhanced/folate-receptor-specific delivery of Ce6 and the efficient PDT of cervical cancer cells. Nanophotosensitizers of the FaPEGbCDseseCe6 conjugates were fabricated using a dialysis process. They showed spherical shapes with particle sizes less than 300 nm. They disintegrated when H_2_O_2_ was added and the particle size distribution changed from a monomodal distribution pattern to a multimodal pattern. The fluorescence intensity and Ce6 release rate also increased with the increase of H_2_O_2_ concentration, indicating that nanophotosensitizers displayed ROS sensitivity. The Ce6 uptake ratio, ROS generation and cell cytotoxicity of nanophotosensitizers were significantly higher than those of Ce6 alone against HeLa cells in vitro. Furthermore, the nanophotosensitizers showed folate-receptor-specific delivery capacity and phototoxicity. The intracellular delivery of the nanophotosensitizers was inhibited by blocking the folate receptors, indicating that they had folate-receptor specificity in vitro and in vivo. The nanophotosensitizers had a higher PDT efficacy and efficiently inhibited the growth of tumor masses of HeLa cells in vivo animal tumor xenograft models. These results show that nanophotosensitizers of FaPEGbCDseseCe6 conjugates are promising candidates for the PDT of cervical cancer.

## Data Availability

The presented in this study are available within the manuscript and the supplementary materials.

## Supplementary Materials

The following are available online at https://www.mdpi.com/article/10.3390/cells10092190/s1. Figure S1: Synthesis scheme and 1H NMR spectra of Ce6-selenocystamine conjugates. Figure S2: Chemical structure and 1H NMR spectra of succinyl β-cyclodextrin, Figure S3: Synthesis scheme and 1H NMR spectra of FA-PEG conjugates, Figure S4: Flow cytometric analysis of HeLa cells treated with Ce6 or nanophotosensitizers. The effect of blocking the folate receptors of HeLa cells. Prior to treatment of Ce6 or nanophotosensitizers, FA (10 mM) was used to pretreat the cells for 30 min to block the folate receptors. For the flow cytometry measurement, cells (1 × 106 cells/well in 6 wells) were treated with Ce6 or nanophotosensitizer (2 μg/mL Ce6 concentration) for 90 min, washed with PBS and treated with Ce6 or nanophotosensitizers. Magnification: ×100. Figure S5: Confocal laser scanning microscopy observation of HeLa cells. Observation: ×400. Figure S6: The effect t of Ce6 alone or nanophotosensitizers on the ROS generation in the absence of light irradiation. ROS generation was measured with DCFH-DA assay. Cells (2 × 10^4^ cells/well in 96 wells) were treated with Ce6 or nanophotosensitizers without irradiation.
