# Supplementary material for: Redox-Sensitive and Folate-Receptor-Mediated Targeting of Cervical Cancer Cells for Photodynamic Therapy Using Nanophotosensitizers Composed of Chlorin e6-Conjugated β-Cyclodextrin via Diselenide Linkage
Source: Cells. 2021 Aug 25;10(9):2190. doi: 10.3390/cells10092190 (PMC8465130; doi:10.3390/cells10092190)
Supplement: Supplementary file 1 [file cells-10-02190-s001.zip › cells-1313265-supplementary.pdf]

Supplementary Materials

Experimental

Materials

Ce6 was purchased from Frontier Sci. Co. (Logan, UT. USA). Folic acid, succinyl β-cyclodextrin, N-(3-dimethylaminopropyl)-N′-ethylcarbodiimide hydrochloride (EDAC), N-hydroxysuccinimide (NHS) and selenocystamine dihydrochloride were purchased from Sigmaaldrich Chem. Co. (St. Louis, Missouri, USA). Poly(ethylene glycol)-diamine (molecular weight: 2000 g/mol) was purchased from SunBio Co. Ltd. (Seoul, Korea).

Synthesis of FaPEGbCDseseCe6 Conjugtes

Ce6-selenocystamine conjugates: Ce6 (478 mg) dissolved in 10 ml DMSO was mixed with equivalent mole of EDAC and NHS and then magnetically stirred for 3 h. To this solution, three equivalent mole of selenocystamine dihydrochloride (762 mg) dissolved in 20 ml DMSO/water mixtures (9/1, *v/v*) was added with two equivalent mole of TEA and then magnetically stirred for more than 12 h. Following this, resulting solution was introduced into dialysis tube (MWCO = 500 g/mol) and then dialyzed against water for 2 days. Water was exchanged every 3 h intervals to remove organic solvents and byproducts. Following this, dialyzed solution was lyophilized over 2 days.

FA-PEG conjugates: 133 mg of folic acid was dissolved in 20 mL of DMSO with equivalent mole of EDAC and NHS. To this solution, 600mg of poly(ethylene glycol)-diamine was added and magnetically stirred for 24 h. Following this, resulting solution was introduced into dialysis tube (MWCO = 2000 g/mol) and then dialyzed against water for 2 days. Water was exchanged every 3 h intervals to remove organic solvents and byproducts. Resulting solution was lyophilized over 2 days to obtain FA-PEG conjugates.

Flow Cytometry

HeLa cells (1×10^6) in 6 well plates were treated with Ce6 or nanophotosenstitizer (2 μg/mL Ce6 concentration) for 90 min. After that, cells were washed with PBS twice and then harvested by trypsinization. This was used to measure with flow cytometry (Invitrogen Attune NxT flow cytometers, ThermoFisher Scientific, Waltham, USA).

Confocal Scanning Microscope

HeLa cells (3×10^5) were seeded in 6 well plates with cover glass and then treated with either the Ce6 alone or nanophotosenstitizers for 90 min. After that, cells were washed with PBS twice and then fixed with 4 % paraformaldehyde for 15 min at room temperature. These were washed with PBS twice and then immobilized with mounting solution (Immunomount, Thermo Electron Co., Pittsburgh, PA, USA). The cells were observed with confocal laser scanning microscope (LSM 800, Carl Zeiss Microscopy GmbH, Jena, Germany).

Results

One of the carboxyl group of Ce6 was activated with EDAC/NHS system and then conjugated with amine group of selenocystamine (Ce6-selenocystamine conjugates). As shown in Figure S1, ^{1}H NMR spectra showed that the specific peaks of Ce6 was confirmed at 1~8 ppm while the ethylene protons of selenocystamine was confirmed at 3.0~3.2 ppm. The Ce6-selenocystamine conjugates showed the specific peaks both the Ce6 and selenocystamine as shown in Figure S1.

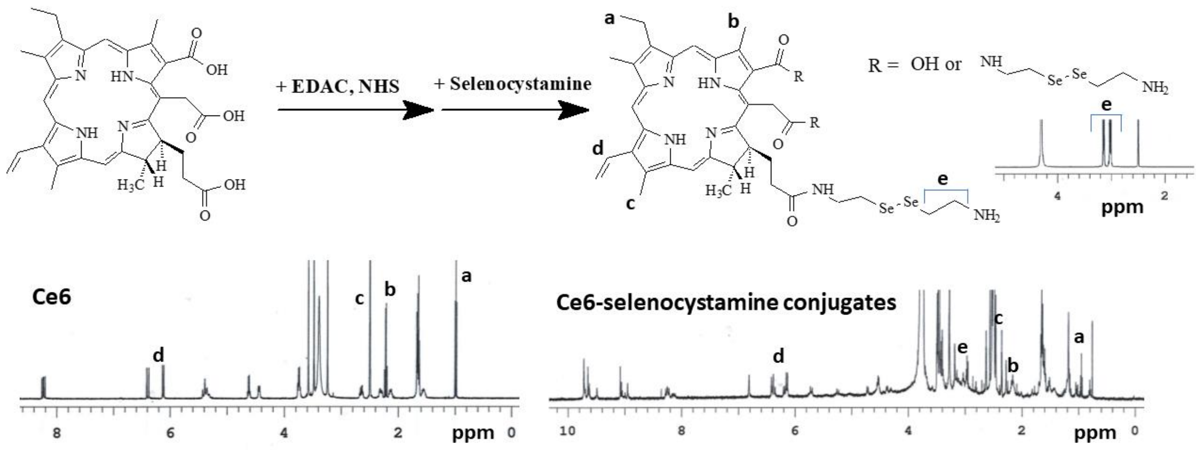

Figure S1. Synthesis scheme and ^{1}H NMR spectra of Ce6-selenocystamine conjugates.

Figure S2 shows the chemical structure and ^{1}H NMR spectra of succinyl β-cyclodextrin. The specific peaks of succinyl β-cyclodextrin was confirmed between 2 and 7 as shown in Figure S2.

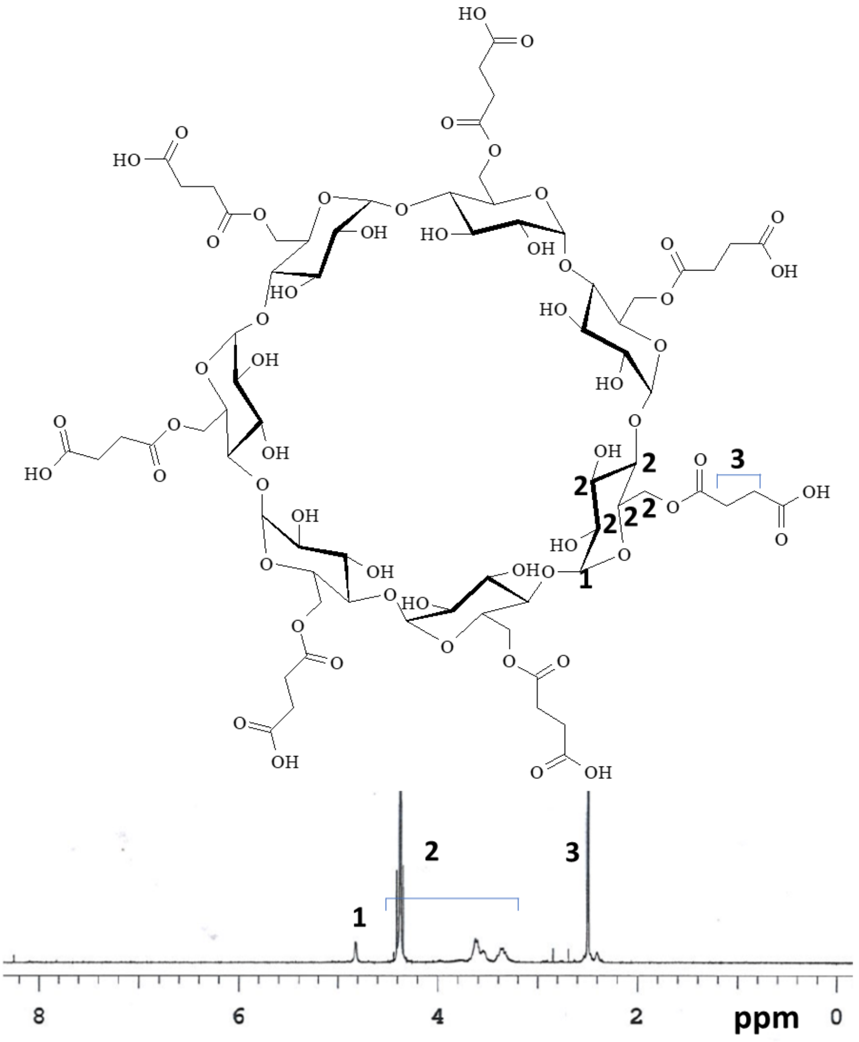

Figure S2. Chemical structure and 1H NMR spectra of succinyl β-cyclodextrin.

Figure S3 shows the synthesis of FA-PEG conjugates. The carboxyl group of FA was conjugated with one of the amine group of PEG and then produced FA-PEG conjugates. The specific peaks of FA were confirmed between 1 and 9 ppm while the ethylene protons of PEG were confirmed at 3.4~3.7 ppm as shown in Figure S3.

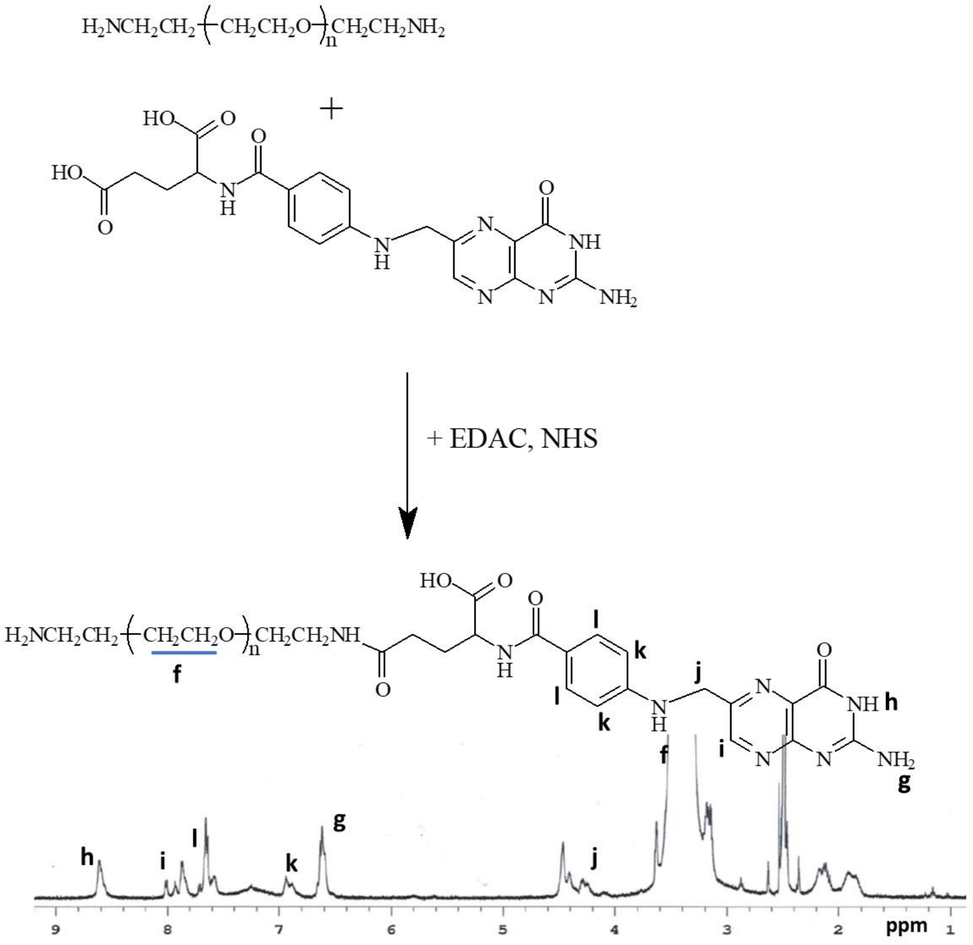

Figure S3. Synthesis scheme and ^{1}H NMR spectra of FA-PEG conjugates.

Figure S4 shows the effect of folate receptor blocking of HeLa cells when the Ce6 alone or nanophotosensitizers were treated. As shown in Figure S4, fluorescence intensity of HeLa cells was increased by the treatment of nanophotosensitizers (FA(−)) and it was significantly changed by blocking the folate receptor (FA(+)). Otherwise, the fluorescence intensity of the treatment of Ce6 was not affected by blocking the folate receptor.

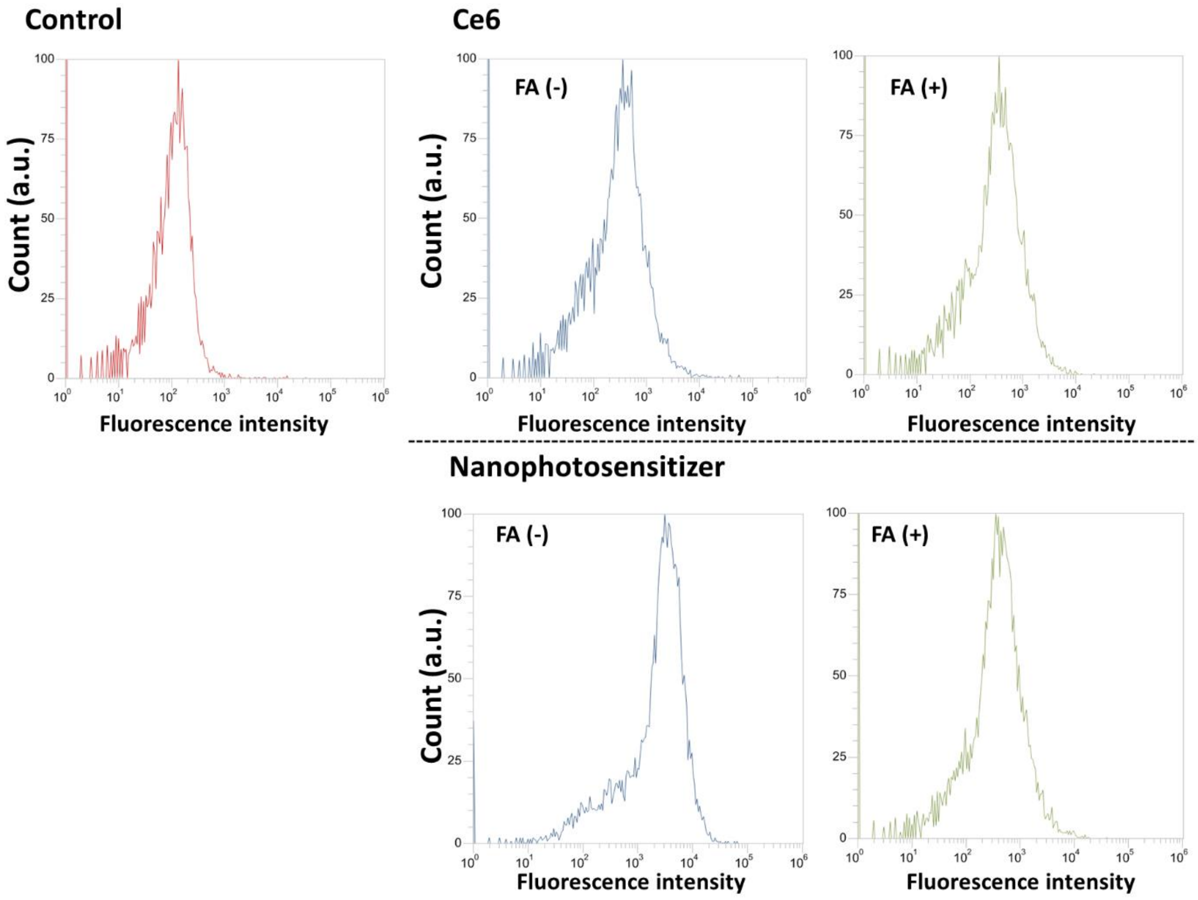

Figure S4. Flow cytometric analysis of HeLa cells treated with Ce6 or nanophotosensitizers. The effect of blocking the Figure 6. or nanophotosensitizers, FA (10 mM) was pretreated to the cells for 30 min to block folate receptor. For flow cytometry measurement, cells (1×10^6 cells/well in 6 wells) were treated with Ce6 or nanophotosenstitizers (2 μg/mL Ce6 concentration) for 90 min and then washed with PBS to measure fluorescence intensity.

Figure S5 shows the confocal scanning microscope images of HeLa cells treated with nanophotosensitizer. Cells were pretreated with the excess amount of folic acid to block folate receptor of HeLa cells and then nanophotosensitizers were treated. In the absence of folate receptor blocking (FA(−)), the red fluorescence intensity of HeLa cells was evident and strong while blocking the folate receptor (FA(+)) significantly decreased red fluorescence intensity in the intracellular compartment of HeLa cells as shown in Figure S5.

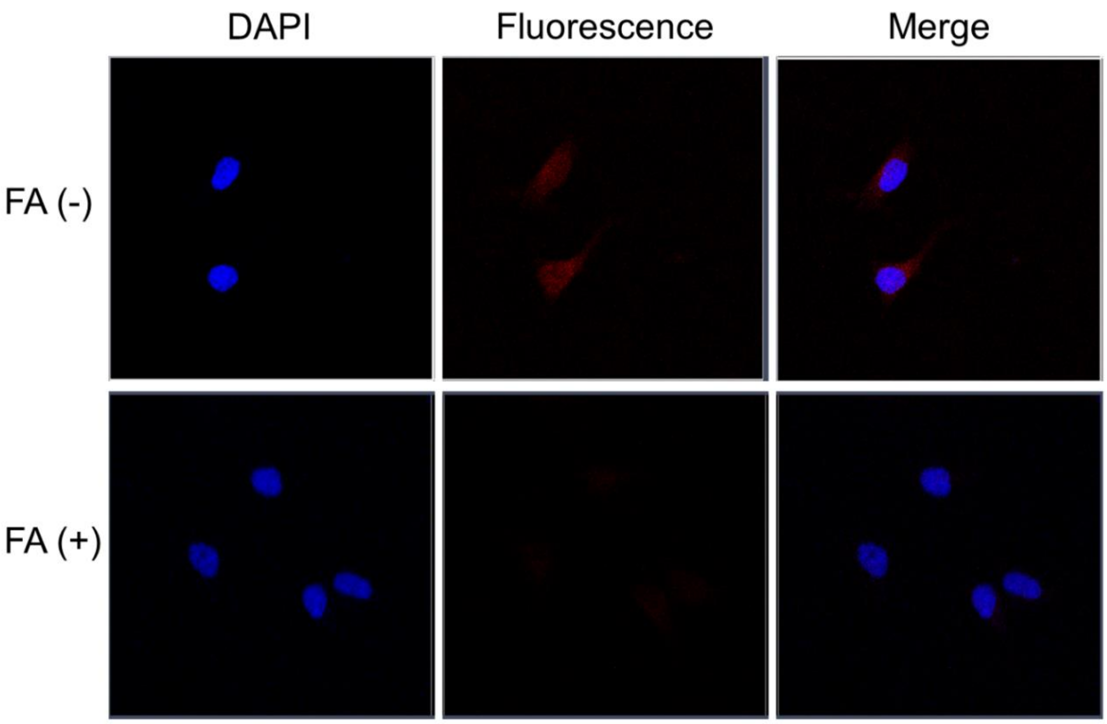

Figure S5. HeLa cells observed with confocal scanning microscope. The cells were pretreated with folic acid as similar to Figure 7 and, after that, the nanophotosensitizers were treated. Magnification: 400×.

Figure S6. shows ROS generation by the treatment of Ce6 alone or nanophotosensitizers. As shown in Figure S6, the ROS generation in HeLa cells was negligible in the absence of light irradiation both the Ce6 and nanophotosensitizers, i.e. ROS level both the Ce6 and nanophotosensitizers was less than 130% at 5 μg/mL Ce6 concentration.

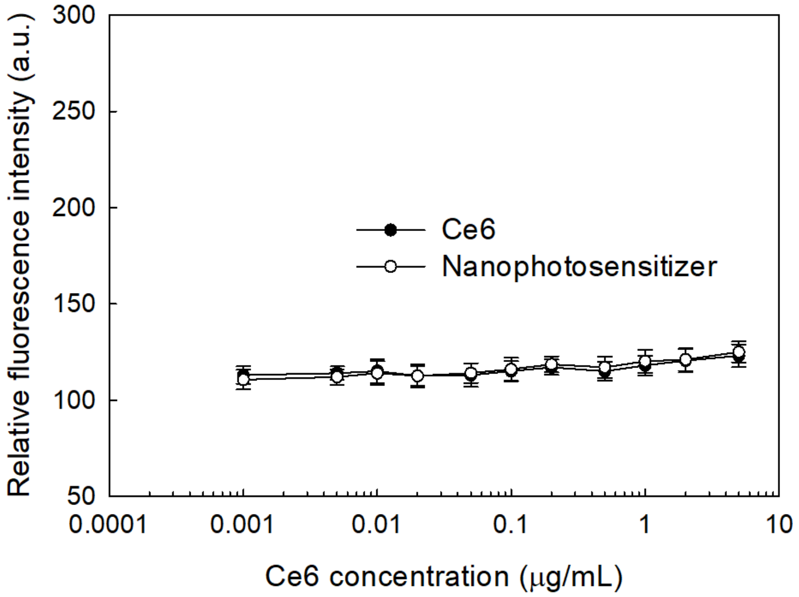

Figure S6. The effect of the Ce6 alone or nanophotosensitizers on the ROS generation of HeLa cells in the absence of light irradiation. The cells (2×10^4 cells/well in 96 wells) were treated with the Ce6 alone or nanophotosensitizers without irradiation. The ROS generation was measured with DCFH-DA assay. All values are average ± S.D. from the results of a single independent experiment with eight replicates.
